# Conversion of human and mouse fibroblasts into lung-like epithelial cells

**DOI:** 10.1038/s41598-019-45195-y

**Published:** 2019-06-21

**Authors:** Amy P. Wong, Sharareh Shojaie, Qin Liang, Sunny Xia, Michelle Di Paola, Saumel Ahmadi, Claudia Bilodeau, Jodi Garner, Martin Post, Pascal Duchesneau, Thomas K. Waddell, Christine E. Bear, Andras Nagy, Janet Rossant

**Affiliations:** 1 Program in Developmental & Stem Cell Biology, SickKids Research Institute, Hospital for Sick Children, Toronto, ON Canada; 2 Program in Translational Medicine, SickKids Research Institute, Hospital for Sick Children, Toronto, Canada; 3 Lunenfeld-Tanenbaum Research Institute, Sinai Health System, Toronto, ON Canada; 4 Program in Molecular Medicine, SickKids Research Institute, Hospital for Sick Children, Toronto, Canada; 5 Latner Thoracic Surgery Research Laboratories, Toronto General Research Institute, and the McEwen Centre for Regenerative Medicine, Toronto, Canada; 6 Department of Molecular Genetics, University of Toronto, Toronto, ON Canada; 7 Department of Obstetrics and Gynaecology, University of Toronto, Toronto, Canada

**Keywords:** Transdifferentiation, Stem-cell biotechnology

## Abstract

Cell lineage conversion of fibroblasts to specialized cell types through transdifferentiation may provide a fast and alternative cell source for regenerative medicine. Here we show that transient transduction of fibroblasts with the four reprogramming factors (Oct4, Sox2, Klf4, and c-Myc) in addition to the early lung transcription factor Nkx2-1 (also known as Ttf1), followed by directed differentiation of the cells, can convert mouse embryonic and human adult dermal fibroblasts into induced lung-like epithelial cells (iLEC). These iLEC differentiate into multiple lung cell types in air liquid interface cultures, repopulate decellularized rat lung scaffolds, and form lung epithelia composed of Ciliated, Goblet, Basal, and Club cells after transplantation into immune-compromised mice. As proof-of-concept, differentiated human iLEC harboring the Cystic Fibrosis mutation dF508 demonstrated pharmacological rescue of CFTR function using the combination of lumacaftor and ivacaftor. Overall, this is a promising alternative approach for generation of patient-specific lung-like progenitors to study lung function, disease and future regeneration strategies.

## Introduction

The high cost of treatments and drug-response variations related to genetic background and environmental differences have made it difficult to find optimal therapies for patients with lung diseases even with the same genetic cause such as in Cystic Fibrosis (CF). Hence, there is a need to develop effective assays to monitor disease phenotype and response to treatments in disease-relevant tissues in a patient-specific manner. Primary airway cells such as basal cells serve as an attractive cell source for disease modeling and therapy discoveries because they often reflect the native tissue and contain highly specialized cell types that are not found in cell lines. Many studies, especially of late^1,2^, have uncovered the multi-lineage differentiation potential of basal cells making these cells an attractive cell source for regenerative medicine. However, there are a few caveats of primary airway basal cells. First, while harvesting primary bronchial basal stem cells is possible, the procedure is invasive making it an unattractive option for sick patients with lung disease. Furthermore, the cells isolated from these patients are commonly infected and results in poor quality cells for expansion *in vitro*. Second, primary basal cells have finite growth potential with very limited expansion ability (often 2–3 passages). As such, re-sampling is often needed from the same patient. This leads to the third issue of batch-to-batch variation in the cells making reproducibility difficult.

Induced pluripotent stem (iPS) cells provide an unparalleled resource for disease modeling and potential cell-based repair. Methods to direct the differentiation of iPS cells into various tissue-specific cell lineages including the lung have enabled their use in CF disease modeling and drug discovery^3–6^. While recent methods have improved the efficiency of generating purified lung epithelial cells *in vitro*^6–8^, a limiting factor in using iPS cells is the laborious, time-consuming and costly nature of generating specialized cell types. Reprogramming somatic cells into iPS cells followed by directed differentiation into lung cells can take anywhere from 6–8 months. To circumvent the iPS stage, studies have shown that overexpression of lineage-specific transcription factors can lead to direct lineage conversion of fibroblasts to neurons^9,10^, cardiomyocytes^11^, intestinal progenitors^12^, renal tubular cells^13^ and hepatocytes^14^. However, many of these direct conversion studies have largely yielded immature, embryonic-like or cell phenotypes of an uncertain relationship to their *in vivo* counterparts.

Overexpression of a combination of the pluripotency factors (OSKM) with and without other lineage-specific factors has also been shown to convert fibroblasts into hematopoietic blood progenitors^15^, endothelial cells^16^, functional cardiomyocytes^17^ and neuronal cells^18^. This approach has led to some controversy over whether this indeed is a direct lineage conversion strategy or occurs via a transient intermediary pluripotent state^19,20^. In either case, the epigenetically unstable state that occurs during the OSKM-mediated reprogramming process^21–24^ seems to allow the cells to respond to appropriate developmental cues and undergo lineage conversion. This is supported by recent studies that show rapid chromatin remodeling enables direct fibroblast reprogramming into neuronal subtypes^25,26^. Use of small molecules to regulate epigenetic modifiers can convert fibroblasts into pancreatic beta cells^27^, functional cardiomyocytes^28^ and neurons^29^.

While direct lineage conversion has been achieved for some endoderm lineages, this has not yet been achieved for the lung. Here, we report the reproducible generation of induced lung-like epithelial cells (iLEC) from human adult dermal fibroblasts and mouse embryonic fibroblasts by transient overexpression of *OCT4*, *SOX2*, *KLF4*, *cMYC* and *NKX2-1* followed by directed differentiation towards lung phenotypes. Mouse iLEC form airway structures in *in vivo* xenotransplants and can repopulate decellularized lung scaffolds with various lung epithelial cell types. Similarly, human iLEC form airway epithelia *in vivo* and differentiate in ALI cultures with measurable functional chloride channel (CFTR) activity. As proof-of-concept, human iLEC-derived epithelia can be used to study drug-induced correction of CFTR function in cystic fibrosis mutant cells. Overall these results indicate that iLEC can be used for drug discovery in lung disease, and with further refinement, iLEC may provide an alternative cell source for tissue regeneration.

## Results

### Generation of mouse iLEC by directed lineage conversion

**Figure 1:**
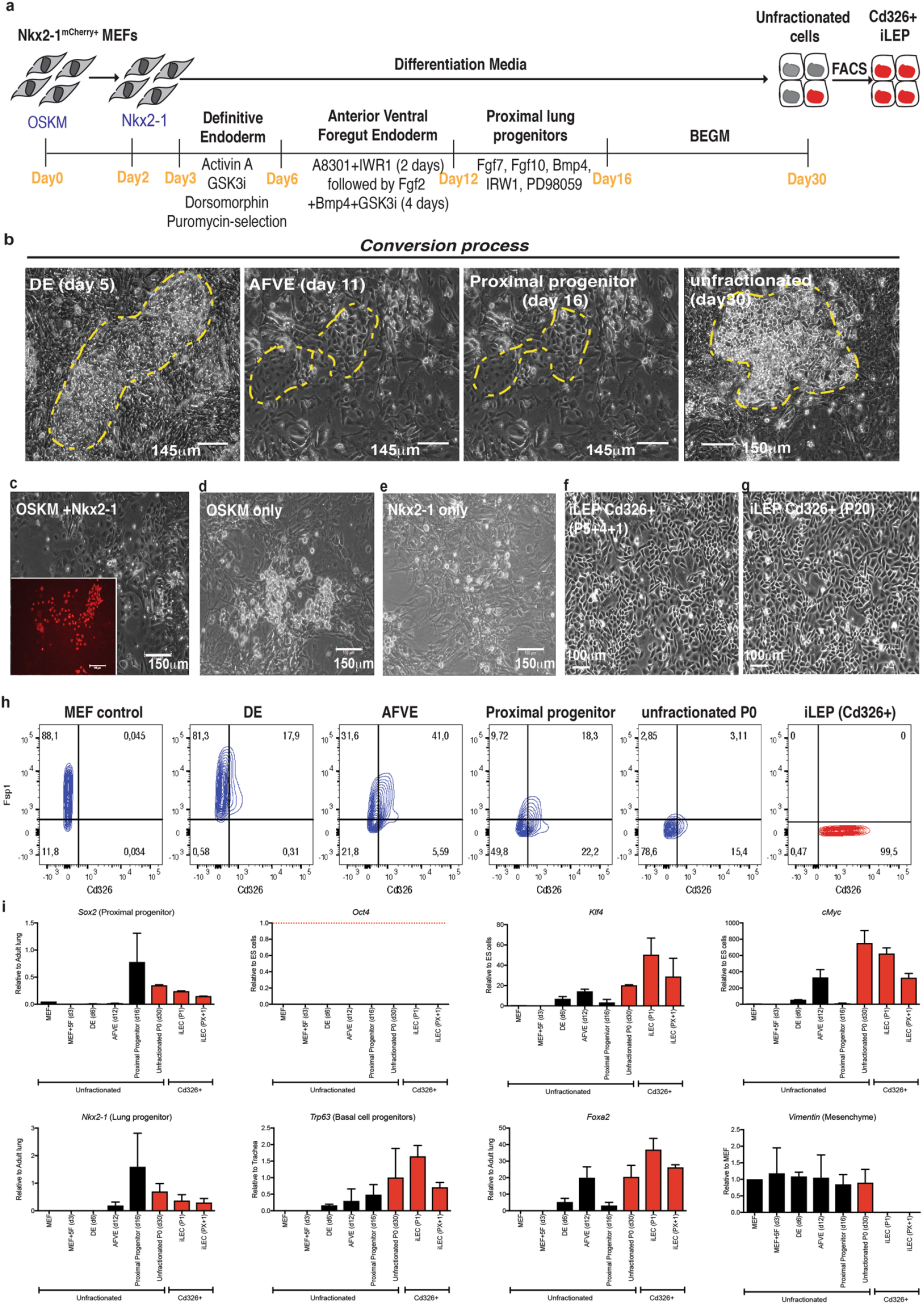
Generation of mouse iLEC by directed lineage conversion. (**a**) Illustrative scheme of the directed lineage conversion process of mouse embryonic fibroblasts from Nkx2-1-mCherry e15.5 embryos into induced lung epithelial progenitors (iLEP). (**b**) Representative DIC images of colonies in various stages of the conversion process. Only fibroblasts transduced with OSKM and Nkx2-1 showed evidence of epithelial-like cells (yellow hatched area). (**c**) At the end of the conversion, epithelial-like cells express nkx2-1-driven mCherry transgene. Note: mCherry fluorescence was relatively dim. This image exposure was increased to show the dim fluorescence. Representative DIC image of (**d**) OSKM only or (**e**) Nkx2-1 only transduced cells showing no morphological change into epithelial-like cells. Representative DIC image Cd326 + FACS-sorted cells (**f**) after freeze-thawed twice and (**g**) maintained in BEGM for 20 passages. (**h**) Flow cytometric contour plots depicting epithelial cell surface marker (Cd326) acquisition and down-regulation of stromal cell surface marker Fsp1 during the cell conversion process. (**i**) Real-time qRT-PCR analysis of pluripotency (Oct4, Klf4, cMyc and Sox2), lung epithelial (Nkx2-1, Trp63, Foxa2) and mesenchymal (Vimentin) gene expression. MEF + 5F represents MEFs transduced with OSKM and Nkx2-1. The cells were harvested 24 hours after Nkx2-1 transduction. DE to iLEC groups represents cells that were transduced with all 5 factors and subsequently differentiated into the respective cell types. N = 4 independent conversion experiments. Scale bars represent 100–150 μm.

Mouse embryonic fibroblasts (MEFs) derived from our Nkx2-1-mCherry knock-in reporter line^30^ were transduced with retroviruses containing the transcription factors Oct4, Sox2, Klf4, cMyc (OSKM) followed two days later by the lung specifying factor Nkx2-1. The cells were then subjected to sequential differentiation cues for 16 days to further drive the differentiation of cells towards lung epithelia as previously described^31^, after which they were maintained and expanded in a commercial medium, BEGM (Fig. 1a, yellow hatched area). While morphological changes were observed as early as 5 days after initiation of definitive endoderm (DE) differentiation, epithelial-like cells only emerged at the anterior ventral foregut equivalent stage (day 11; yellow hatched area, Fig. 1b). These groups of cells expanded into colonies (3–10 epithelial colonies per 10^4^ cells representing a range of 0.03–0.1% conversion efficiency). By the end of the conversion process (day 30; yellow hatched area), some of the cells in the epithelial-like clusters showed mCherry fluorescence suggestive of lung epithelial identity (26) (Fig. 1c). Cells transduced with OSKM, or Nkx2-1 alone did not result in morphological changes resembling epithelial phenotypes (Fig. 1d,e) and no mCherry transgene fluorescence was detected. Due to the relatively dim mcherry fluorescence (Supplementary Fig. 1a,b) and the poor cell survival following cell sorting of the rare mCherry+ cells, we chose to use pan-epithelial cell surface marker Cd326 (Epcam) at the end of the conversion (day 30) to sort for cells with cuboidal epithelial-like cell morphology. These cells could be serially passaged, maintain their phenotype following cryopreservation in liquid nitrogen and subsequent thawing and be maintained in BEGM over time without morphological changes or reversion to fibroblast-like phenotype (Fig. 1f,g). These Cd326+ cells were subsequently called induced lung epithelial-like cells (iLEC). Assessment of chromosomal stability show 75% of the Cd326+ cells show a normal karyotype with 40 chromosomes as assessed by G-banding analysis (Supplementary Fig. 1c). FACS characterization of the cells during the conversion process for epithelial (Cd326) and mesenchymal (Fsp1) markers show a gradual shift towards gain of Cd326 and a concomitant loss of Fsp1 expression (Fig. 1h). Analysis of gene expression during the conversion process demonstrated a gradual up-regulation of lung lineage-related genes (*Nkx2-1*, *Trp63*, *and Foxa2*) (Fig. 1i). High levels of *Trp63* expression were maintained in the iLEC fraction, while the mesenchyme gene *Vimentin* was undetectable in iLEC. While genes associated with pluripotency, *Sox2*, *Klf4* and *cMyc*, were upregulated during the conversion process, *Oct4* (*Pouf51*) was not. To exclude the possibility that non-lung lineages were formed since *Nkx2-1*, *Trp63* and *Sox2* are not exclusive markers of the lung epithelium, genes associated with forebrain (*Pax6*), thyroid (*Tg*, *Pax8*), and liver (*Hnf4*) were assessed in iLEC. Relative to levels in the appropriate control tissue, none of these lineage markers were detected at comparable levels (Supplementary Fig. 1d).

Analysis of the transgene and endogenous gene expression of the reprogramming factors showed that while iLEC expressed high levels of the *Klf4* transgene, the cells did not express high levels of exogenous *Sox2*, *cMyc* and *Oct4* (Supplementary Fig. 1e). Instead, iLEC showed up-regulated expression of endogenous *Nkx2-1* (*Ttf1*), *Sox2*, *cMyc*, *Klf4* but not *Oct4*.

Conversion of the cells without going through the entire differentiation stages (up to day 30) did not improve the iLEC conversion efficiency (Group B, Supplementary Fig. 2a–c). Furthermore, overexpression of Nkx2-1 at different time points along the differentiation process did not improve the conversion of fibroblasts into epithelial-like cells. Instead, the cells could not be maintained (Groups A, C, D), underwent spontaneous epithelial-mesenchymal transition (EMT, Groups E and F), or appeared epithelial-like but expressed pluripotency factor transgenes (Group B, Supplementary Fig. 2d, blue bar).

### Mouse iLEC contain a mixed population of cells including a subset of basal epithelial progenitor cells

**Figure 2:**
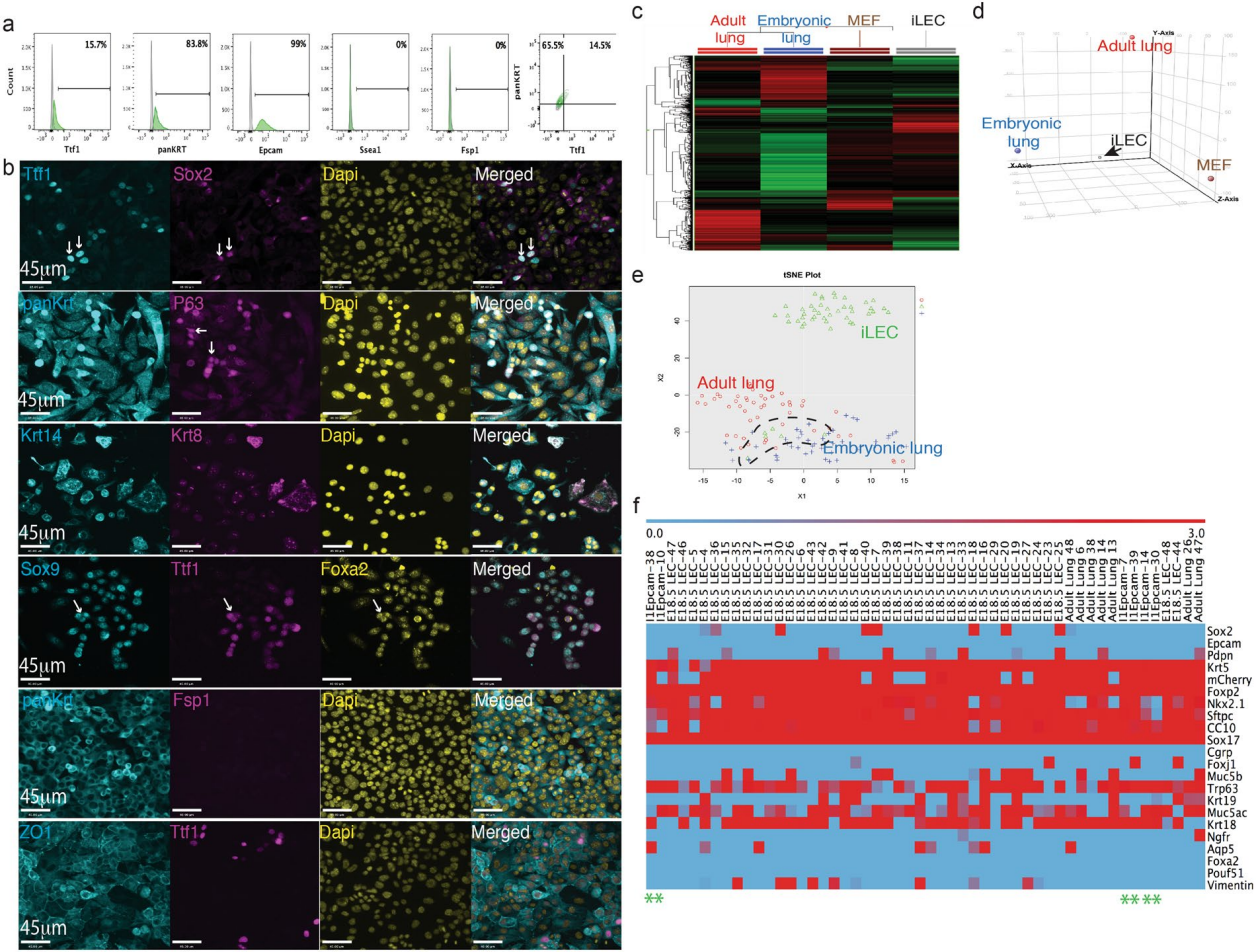
Mouse iLEC characterization. (**a**) Characterization of iLEC by flow cytometry show a small fraction ~15% of the epithelial cells express lung transcription factor Nkx2-1. (**b**) Immunofluorescence characterization of mouse Cd326+ iLECs. Scale bar represents 45 μm. White arrows point to positive cells. (**c**) Unsupervised hierarchical clustering shows global gene expression of mouse iLEC distinct from adult and embryonic lung cells and some distinct expressions compared to parental MEF. (**d**) Principal component analysis shows mouse iLEC segregated from all three control cell types (adult and embryonic lung epithelial cells and parental MEF). (**e**) Principal component analysis of single cells from iLEC, Cd326+ adult lung cells and mcherry-expressing embryonic (e18.5) lung cells (Blue dots represents e18.5 lung cells, red dots represent adult lung cells and green dots represent iLEC). (**f**) Heatmap of the lung genes expressed in 6 single iLEC that clustered with control embryonic mcherry+ and adult CD326+ lung epithelial cells. Green asterisk mark the column for each iLEC.

Flow cytometric analysis of the purified Cd326 + iLEC showed that the majority of the cells expressed epithelial pan-cytokeratins (Fig. 2a). No detectable expression of the pluripotent marker, Ssea1, or fibroblast surface protein, Fsp-1, was found. However, only a fraction (15%) of the iLEC expressed Nkx2-1. Immunofluorescence characterization of the iLEC revealed that all Nkx2-1 iLEC expressed pan-cytokeratin and the tight junction protein Zo1 (Fig. 2b and Supplementary Fig. 3a). Cells co-expressing Nkx2-1 and the proximal lung marker Sox2 as well as the distal epithelial progenitor marker Sox9 were also found suggesting the generation of both proximal and distal lung epithelial-like progenitors (Fig. 2b, white arrows). The P63 protein (or *Trp63* gene, a transcription factor expressed in basal cell progenitors^32^) was expressed in a subset of the iLEC cells suggesting that the iLEC might contain basal cell progenitors. Both single positive Krt14+ and double positive Krt8/18 + Krt14+ expressing cells were also observed. Co-expression of Krt8/18 and Krt14 has previously been shown to mark a transient basal cell progenitor population that is activated upon airway injury and can give rise to ciliated and secretory cells to regenerate the airway epithelium^33,34^. Proteins expressed in mature distal epithelial cells such as Club (Ccsp) and Type II alveolar cells (proSPC) were not detected in mouse iLEC (Supplementary Fig. 3b).

Microarray analyses of mouse iLEC at the population level showed a global transcriptional profile diverging from parental fibroblasts but also distinct from adult lung and embryonic day 18.5 lung (Fig. 2c). Epcam staining and mCherry fluorescence were used to sort epithelial cells from the adult and embryonic lungs respectively as controls. Unbiased principal component analyses showed that the mouse iLEC clustered separately from all three control cell types (Fig. 2d).

Since rare complete conversion events could be masked at the population level, we employed single cell multiplex qPCR using the Fluidigm Biomark HD system to determine conversion efficiency and the heterogeneity of the converted cells. Not surprisingly, principal component analysis of single iLECs revealed that the majority of the cells clustered separately from adult lung epithelial cells and embryonic day 18.5 lung epithelial cells (Fig. 2e**)**. Hierarchical clustering revealed that only 6/48 cells (black hatched/box and denoted on the heatmap with green asterisk) clustered with control lung cells (Fig. 2f and Supplementary Fig. 4). These cells expressed comparable levels of many lung genes including *CC10*, *Sftpc*, *Trp63*, *Krt18*, *Muc5ac*, *Foxp2*, *Nkx2-1*, *Sox17*, *Krt5*. However, the majority of the mouse iLEC (38/48 cells, green box) co-expressed many other genes associated with foregut development (*Foxg1*, *Pax9*, *Otx2*, *and Gata6*) and early stromal cell lineage (*Snail)*, suggesting an incomplete conversion of the fibroblasts towards lung in these cells. Overall, single cell analysis revealed cellular heterogeneity of the mouse iLEC and multi-lineage differentiation during the conversion process.

### Mouse iLEC repopulate decellularized lungs

**Figure 3:**
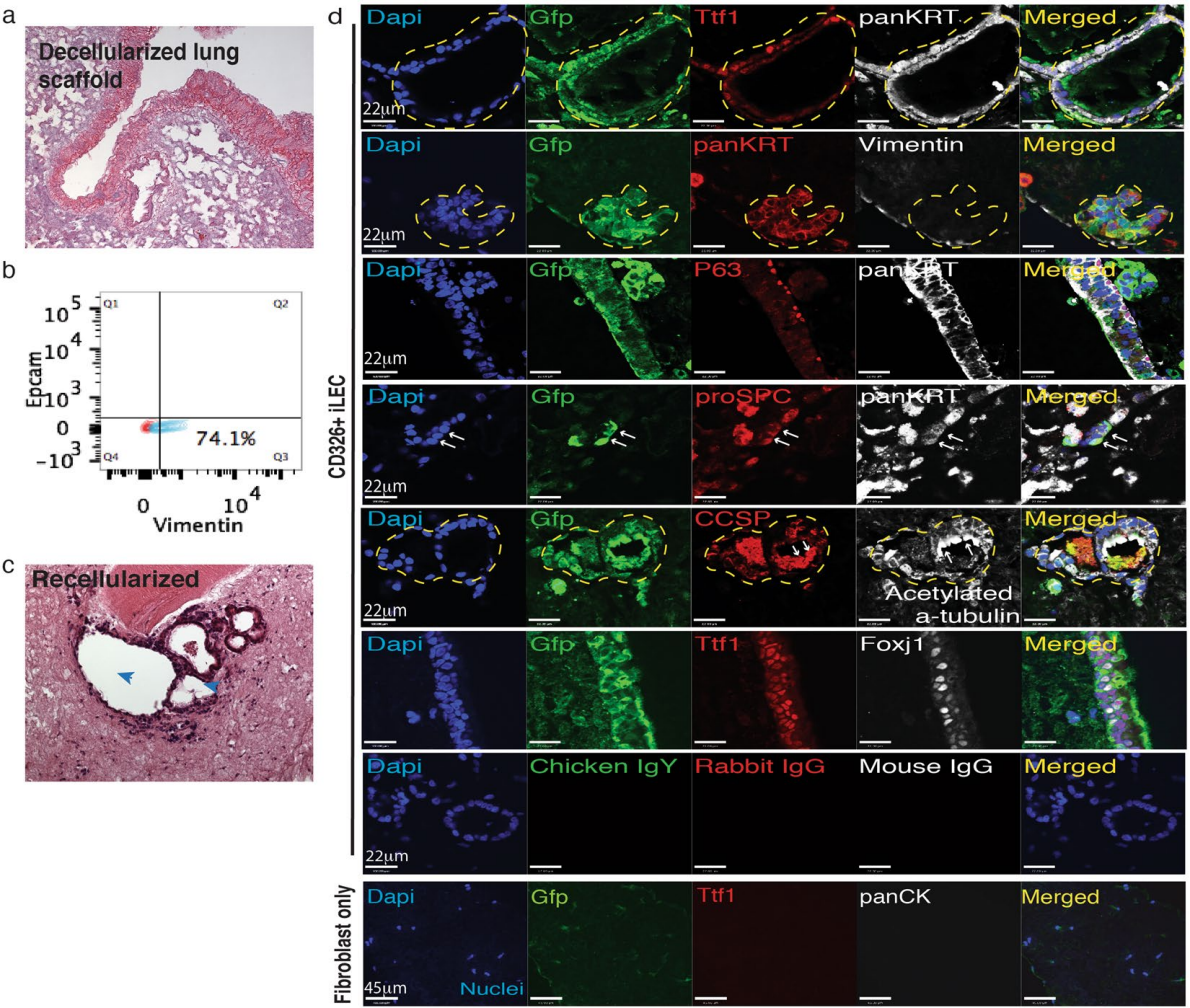
Lung repopulation potential of mouse iLEC. (**a**) Hematoxylin and eosin staining of a rat decellularized lung scaffold. (**b**) Flow cytometric analysis of rat embryonic lung fibroblasts (RELF) for Epcam, epithelial and vimentin, stromal cell markers. (**c**) Recellularized lungs with iLEC and RELF to provide paracrine support show airway reconstitution and evidence of cilia (blue arrowheads) protruding into the luminal space (right). (**d**) Immunofluorescence characterization of the recellularized scaffolds show donor mouse iLEC (Gfp+) reconstituting the airway structures with lung (Nkx2-1+) epithelial (panKrt+) cells. Further characterization shows Club (Ccsp+), Ciliated (Acetylated tubulin+, Foxj1+), Basal (P63+), and cells expressing Type II alveolar epithelial cell marker proSPC+ (white arrows). Of note, pan cytokeratin-expressing Gfp+ cells do not express mesenchymal marker Vimentin. No airway epithelial reconstitution was observed when scaffolds were seeded with rat embryonic lung fibroblasts only. Respective non-immune immunoglobulins were used for staining controls. N ≥ 3 recellularization experiments with separate batches of mouse iLEC, RELF and decellularized scaffolds. Scale bars represent 22–45 μm. Yellow hatched indicate GFP+ iLEC areas co-expressing lung markers.

Given the heterogeneity of the iLEC in culture, we wanted to test whether exposure to lung decellularized scaffolds could drive further differentiation into lung phenotypes. To test the potential of mouse iLEC to form airway structures, decellularized rat lungs were used as biomaterial scaffolds for the cells to engraft. Lack of hematoxylin nuclear staining of the decellularized lungs confirms the absence of native lung cells in the scaffolds (Fig. 3a). Rat embryonic lung fibroblasts (RELF) were used as supporting cells for the recellularization with iLEC. These rat fibroblasts were confirmed by flow cytometry to be vimentin-positive and Epcam-negative, indicating there was no contamination with rat epithelial cells (Fig. 3b). To distinguish donor iLEC from RELF, iLEC were co-transfected with two piggyBac expression vectors: one containing a strong synthetic CAG promoter (CMV enhancer, chicken beta-actin promoter)-driven GFP and another containing the transposase to facilitate recombination. The cells were then sorted for GFP expression prior to recellularization. After 21 days of co-culturing iLEC and RELF on decellularized lung scaffolds, recellularized airway structures with ciliated cells were observed (Fig. 3c, blue arrowheads and Supplementary Fig. 5a, low magnification view of the graft). Donor (GFP+) iLEC recellularized the scaffold with TTF1 and pan-cytokeratin (panKrt) positive cells (Fig. 3d and Supplementary Fig. 5a). In addition, GFP+ cells that were not Nkx2.1+ but panKrt+ were also observed. Donor GFP+ cells formed airway structures containing cells expressing markers associated with Club cells (Ccsp), Ciliated cells (acetylated tubulin and Foxj1), Basal (P63), and Type II alveolar-like cells (proSPC) lineages. To further confirm that it is indeed donor iLEC that contributed to the airway structures, RELF only controls were cultured on the same batch of decellularized lung scaffolds in parallel (Fig. 3d, Fibroblast only group). No repopulation of airway epithelial cells was observed in these control groups and overall engraftment of these cells was minimal. Recellularization with incompletely converted cells (Group B, highest mCherry fluorescence) showed no airway reconstitution potential and instead formed masses of Nkx2-1 expressing non-epithelial cells (Supplementary Fig. 5b,c hatched area) reminiscent of lung tumors^35^.

### Mouse iLEC generate airway structures in ectopic transplants

**Figure 4:**
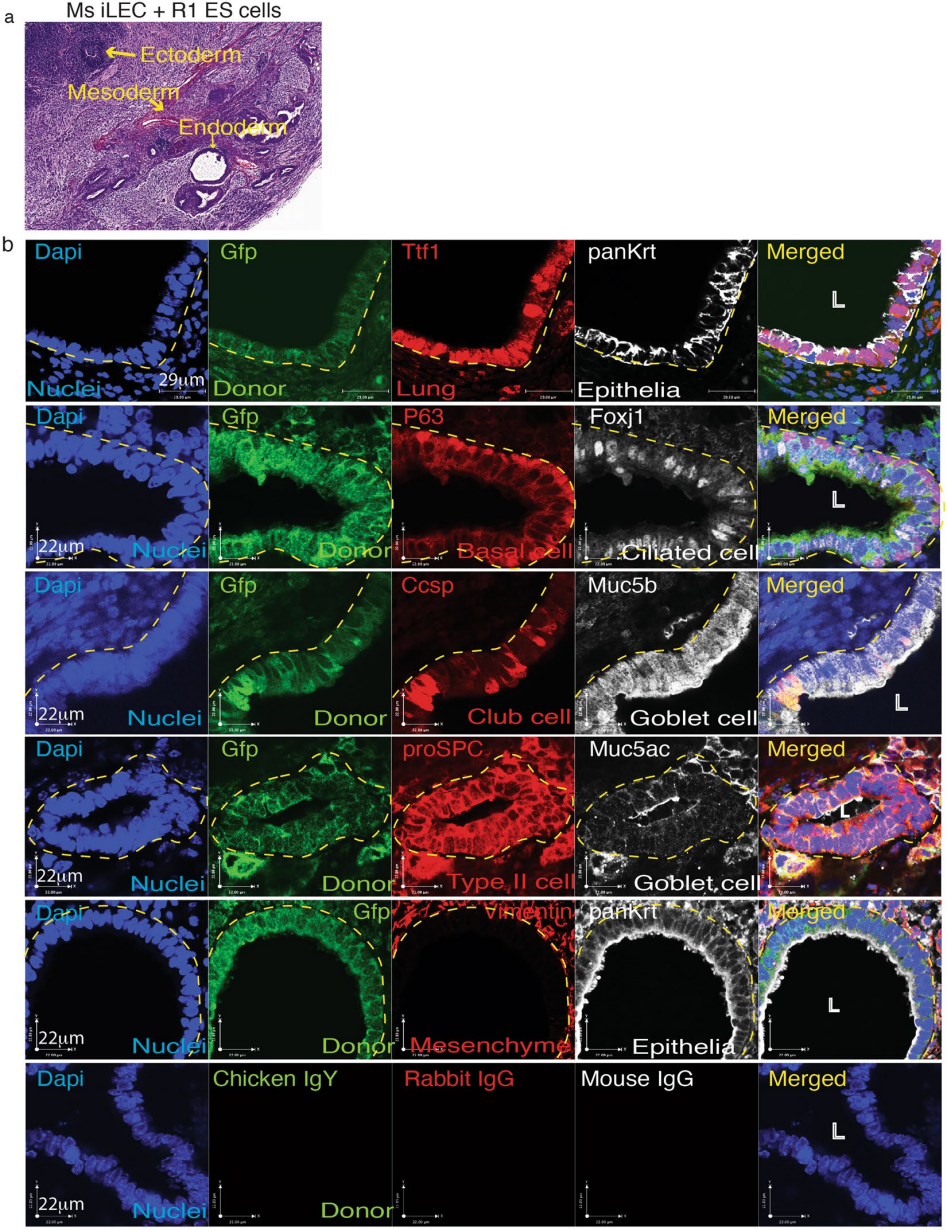
Mouse iLEC form lung epithelia *in vivo*. Mouse iLEC were co-transplanted subcutaneously into NOD-SCID animals with R1 mouse embryonic stem (mES) cells. (**a**) Hematoxylin and eosin staining show teratomas containing all three germ layers, ectoderm, mesoderm and endoderm lineages (yellow arrows). (**b**) Immunofluorescence characterization of the teratomas demonstrates presence of donor iLEC-derived (GFP+) lung (Ttf1+) epithelium (panKrt+). Basal (P63+), Ciliated (Foxj1+), Club (Ccsp+), Goblet (Muc5ac+, Muc5b+) and Type II alveolar cell (proSPC+) were found to line the iLEC-derived epithelium. White arrows point to positive cells. White arrow points to iLEC-derived GFP+ cells contributing to the supporting mesenchyme (Vimentin+). N = 6 teratomas from 3 mice using three separate batches of mouse iLEC, L; lumen. Scale bars represent 22–29 μm.Yellow hatched indicate GFP+ iLEC areas co-expressing lung markers.

To determine whether mouse iLEC can generate lung epithelial structures in an *in vivo* teratoma assay, iLEC and R1 mouse embryonic stem (ES) cells (4:1 ratio) were co-transplanted subcutaneously into the fat-pad of immunodeficient mice. This assay challenges the multi-lineage contribution of the iLEC in a permissive environment where cells of all three germ layers could form. Prior to transplantation, mouse iLEC were transfected with a piggyBac-mediated transgene expressing constitutive GFP. Mouse R1 ES cells alone formed teratomas within 4 weeks (Supplementary Fig. 6a). However, transplantation of 10^7^ mouse iLEC alone or the parental mouse embryonic fibroblasts (MEFs) alone did not form any cell/tissue mass that could be detected and harvested after 10 weeks (data not shown). Mouse iLEC co-transplanted with mouse embryonic stem cells (4:1 ratio) formed teratomas containing ectoderm, mesoderm and endoderm tissues (Fig. 4a). Both ES cell-derived (GFP−) and ES/iLEC-derived (GFP+) teratomas formed lung epithelial structures with cells representing basal (P63+), ciliated (Foxj1+), Club (Ccsp+), surface airway epithelia (Muc5b+) and Goblet (Muc5ac+) cell lineages (Fig. 4b and Supplementary Fig. 6b). Pro-surfactant protein-C was also expressed in donor-derived epithelial cells suggesting the formation of distal airway epithelium. GFP+ stroma (yellow arrow) was also observed suggesting iLEC could also contribute to the surrounding stroma of airways. Mouse lung tissues were used as staining controls in addition to non-immune isotype controls (Supplementary Fig. 7).

### Generation of human iLEC by directed lineage conversion

**Figure 5:**
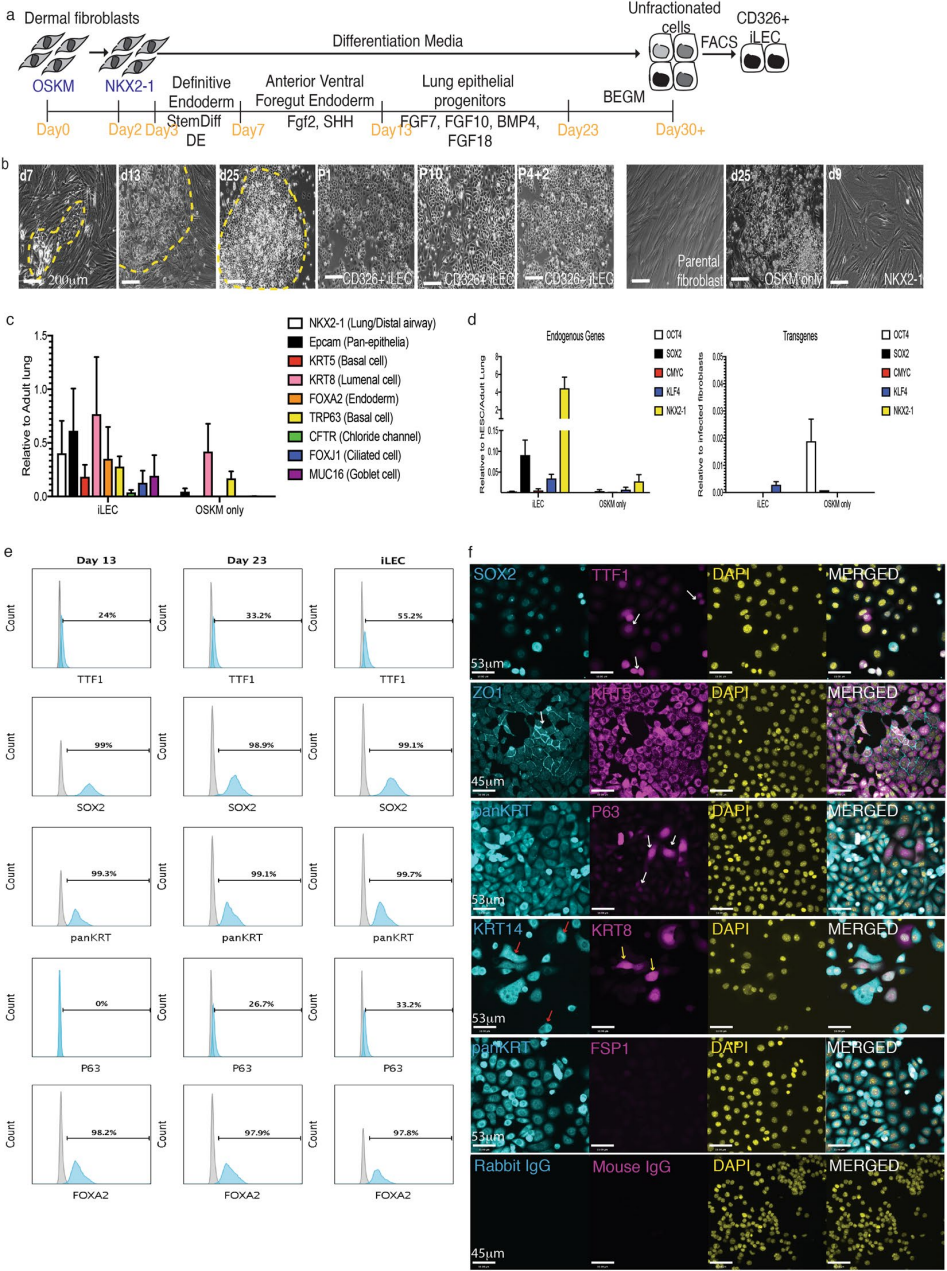
Human iLEC conversion and characterization. (**a**) Schematic of the directed lineage conversion process of human dermal fibroblasts into iLEC. N = 4 cell lines converted. (**b**) Representative DIC images of epithelial-like colonies throughout the conversion process (yellow hatched). FACS-sorting for epithelial cell surface protein CD326 revealed homogenous morphologies (P1) in culture that could be expanded for multiple passages (P10) and after freeze-thaw (P4 + 2). Representative images of parental fibroblast, cells transduced with OSKM or Nkx2-1 alone show no evidence of epithelial like cells by the end of the conversion process. (**c**) Gene expression analysis of iLEC of several lung epithelial cell markers. (**d**) Assessment of endogenous and transgene expression of the reprogramming factors used in the conversion. Error bars represent SEM on characterization of four human iLEC lines. (**e**) Representative flow cytometric characterization of the cells at day 13 (AFVE), day 23 (Lung progenitor) and iLEC stages. (**f**) Representative immunofluorescence characterization of human iLEC show these cells express epithelial cell markers. Scale bars represent 53–200 μm

Human dermal fibroblasts were first infected with a lentivirus encoding a mouse ecotropic retroviral receptor (Slc7a1 gene) to make the human cells receptive to retroviral infection as previously described^36^. These cells were then transduced with mouse retroviruses encoding the human transcription factors OSKM. Two day later, the cells were transfected with a plasmid containing the cDNA for the lung-specifying factor NKX2-1. The cells were then subjected to sequential differentiation cues to generate lung progenitors as we have previously described^4^. After day 23, the cells were maintained and expanded in commercial BEGM medium (Fig. 5a). Patches of epithelial-like morphology (yellow hatched area) were observed as early as 7 days into the conversion process (Fig. 5b and Supplementary Fig. 8a) that expanded over the course of the conversion process. Consistently from 4 human fibroblast lines, 5–15 epithelial-like colonies per 1 × 10^5^ seeded cells emerged from the conversion representing a conversion efficiency range from 0.005–0.01% from 3 independent reprogramming events. To enrich for epithelial cells, FACS sorting for the epithelial cell surface marker CD326 was performed, as with the mouse cells. The CD326+ sorted fraction was subsequently called iLEC. CD326+ cells represented 0.8–10% of the cells at the end of the conversion process. CD326+ human iLEC could be expanded and freeze-thawed for at least 10 passages while maintaining the epithelial morphology in culture (Fig. 5b). Importantly, cells transduced with OSKM alone showed some morphological changes but further analysis of these cells demonstrated that they did not exhibit lung phenotypes (Fig. 5b,c). Cells transfected with NKX2-1 alone did not show any morphological changes while non-transduced cells did not survive in the differentiation media. Gene expression profiling showed low but detectable expression of several airway epithelial markers (*NKX2-1*, *KRT5*, *FOXJ1*, *TRP63* and *MUC16*) in human iLEC (Fig. 5c). Analysis of the iLEC cells post-sort showed up-regulated expression of endogenous *NKX2-1*, *SOX2* and to a lesser extent *KLF4* and *cMYC*, compared to cells converted with OSKM alone which expressed high levels of the OCT4 transgene (Fig. 5d). No significant expression of the exogenous transgenes was detected in the final iLEC cultures. CD326 + iLEC also expressed pan-cytokeratins (panKRT), SOX2 and FOXA2 (over 95%) while a large fraction of the cells (55%) were also positive for NKX2-1 (Fig. 5e). The iLEC population contained ~30% cells that expressed the basal progenitor cell marker P63.

Immunofluorescence confirmed co-localization of NKX2-1 and SOX2 in a subset of iLEC (Fig. 5f). Meanwhile staining for P63 was present in a small subset of the iLEC epithelial cells (panKRT+). Human iLEC were positive for expression of the tight junction protein ZO1+ and, similar to mouse iLEC, contained KRT8/18 + KRT14+ double positive cells (yellow arrow) as well as KRT14+ cells (red arrows). Human iLEC do not express the fibroblast cell surface protein (FSP1). Human bronchial epithelial cells were used as staining controls (Supplementary Fig. 8b).

### Human iLEC generate lung epithelium in ectopic transplants *in vivo*

**Figure 6:**
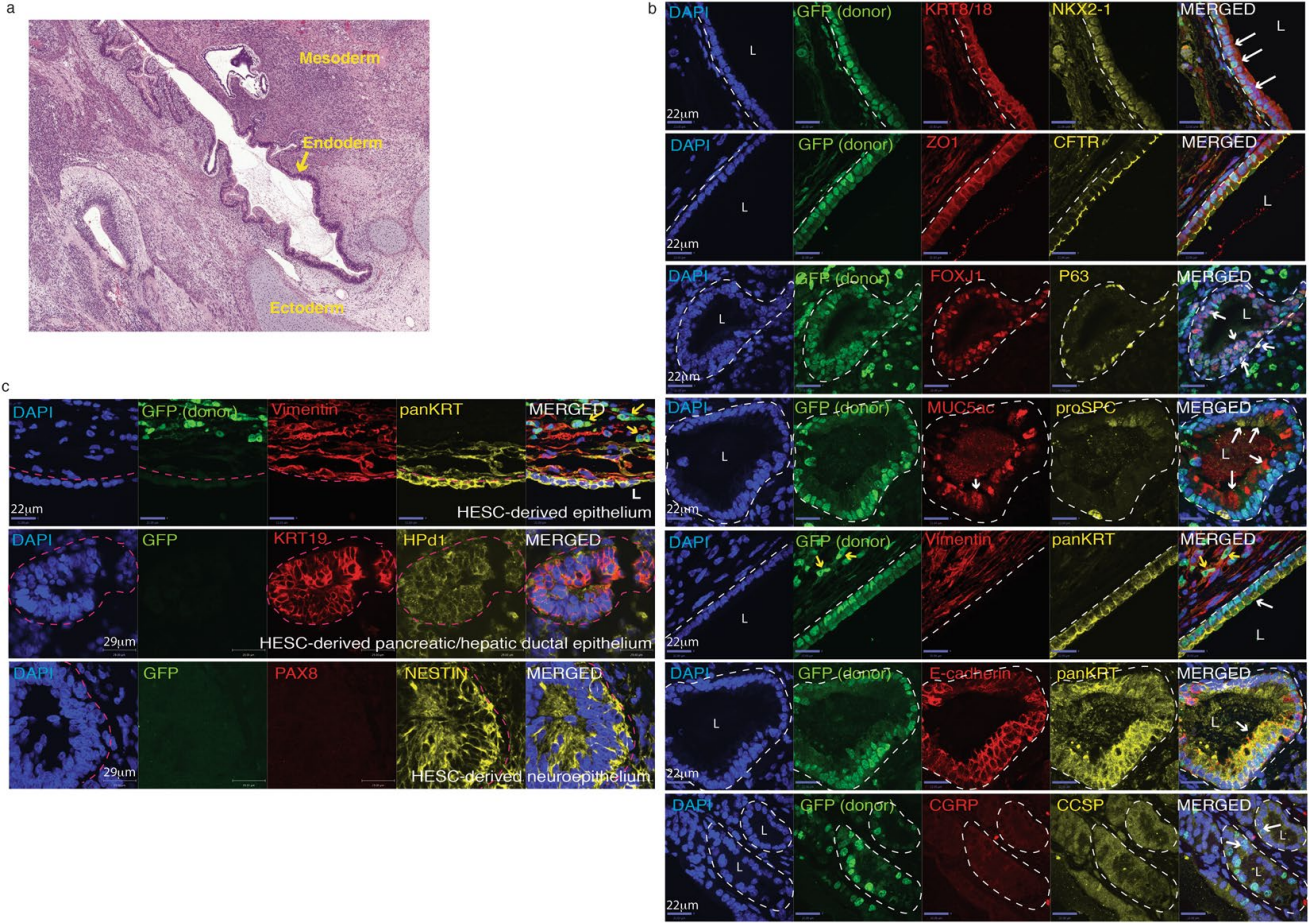
Human iLEC form lung epithelia *in vivo*. Human iLEC were co-transplanted subcutaneously into NOD-SCID animals with human embryonic stem cells. (**a**) Hematoxylin and eosin staining show teratomas containing all three germ layers, ectoderm, mesoderm and endoderm lineages. (**b**) Immunofluorescence characterization of the teratomas demonstrates presence of human iLEC-derived GFP+ lung epithelial cells expressing NKX2-1 and KRT8/18. Ciliated (FOXJ1+), Basal (P63+), Goblet (MUC5ac+) and Type II alveolar cell (proSPC+) were found lining the iLEC-derived epithelium. Apical expression of CFTR was also detected. White hatched represent GFP+ iLEC areas co-expressing specified markers.White arrows point to positive cells. Yellow arrows point to iLEC-derived (GFP+) mesenchyme (Vimentin+). (**c**) High magnification of hepatic/pancreatic ductal tissue (KRT19 + Hpd1+, white hatched area) and neuronal (Nestin+) cell clusters were human ES cell-derived. Donor (GFP+) stromal cells (Vimentin+) surround human ES-derived epithelial (panKRT + GFP−). Pink hatched represent GFP− hES-derived areas co-expressing specified markers.Teratoma experiments were replicated n = 2 times with four human iLEC lines. L; lumen. Scale bars represent 22–29 μm.

To determine whether human iLEC can generate lung epithelial structures *in vivo*, human iLEC were transplanted subcutaneously into immunodeficient mice. Prior to transplantation, human iLEC were transfected with a piggyBac-mediated transgene expressing constitutive nuclear GFP. Human iPS and ES cells formed teratomas within 5-8 weeks, while transplantation of 10^7^ human iLEC did not form a tumor and the mice were sacrificed 16 weeks after cell transplantation. No evidence of any cell mass was observed (data not shown). Human iLEC were then co-transplanted with human ES cells (1:4 ratio, ES: iLEC) to challenge the iLEC in an assay that favors multi-lineage differentiation. Teratomas containing ectoderm, mesoderm and endoderm lineages were observed in human iLEC/ES (Fig. 6a). Donor-derived GFP-positive human iLEP formed airway-like structures containing many airway epithelial cell lineages (Fig. 6b). Characterization of these airway structures shows presence of lung epithelial cells ((NKX2-1 + KRT8/18+, E-cadherin + panKRT+) that also formed tight junctions (ZO1+) and expressed apical CFTR. Expression of specific lung cell type markers were also observed including, Ciliated cells (FOXJ1+, acetylated alpha-tubulin+), Club cells (CCSP+), Basal (P63+), Goblet (Muc5ac+) and Type II alveolar cells (proSPC+) (Fig. 6b and Supplementary Fig. 9a, white arrows). It is unclear whether the cells expressing proSPC represent a rare subset of Club cells^37^ that express this marker or are Type II alveolar cells. No Type I alveolar cells or CGRP+ pulmonary neuroendocrine cells were found. Epithelia derived solely from GFP negative human ES cells could also be found surrounded by GFP+ donor stroma (Vimentin+) (yellow arrows). Clusters of donor cells (GFP+) were observed in OSKM only cell transplants but these cells did not express lung NKX2-1, or epithelial (panKRT) markers (Supplementary Fig. 4a, yellow arrows). To determine whether human iLEC were capable of generating other endoderm and ectodermal lineages, serial sections of the teratomas were stained for liver (albumin), gut (CDX2), thyroid (TG), pancreas (PDX1, HNF6), hepatic/pancreatic ductal epithelium (KRT19, Hpd1), neuroectoderm (PAX6, Nestin) markers (Fig. 6c and Supplementary Fig. 9b). Human iLEC did not contribute to any of these lineages. Rather cells that were positive (yellow arrows or white hatched area) for albumin, PDX1, PAX6, KRT19 and Hpd1 were not donor-derived (GFP−) and therefore must be human ES-derived. Not surprisingly, human ES-derived airway epithelium was found in teratomas derived solely from human ES cells (Supplementary Fig. 10a). Human tracheal and lung tissues were used as staining controls (Supplementary Fig. 10b). Interestingly, supporting GFP+ cells also appeared to surround the GFP+ epithelium (Fig. 6c, yellow arrows) suggesting that donor iLEC may also contribute to the supporting mesenchyme possibly through epithelial-mesenchymal transition. The mechanisms by which human ES cells support the formation of iLEC-derived airway structures is unclear. Possible explanations may be through differentiation into cells that lay down extracellular matrices for the human iLEC to engraft or through cross talk between human ES-derived mesodermal and iLEC that is necessary to support airway formation. Importantly, in an assay that challenges the multi-lineage differentiation potential of the iLEC, the differentiation was restricted to lung epithelia and supporting stromal cell lineages.

### *In vitro* differentiated human iLEC can be used to model CF phenotype

**Figure 7:**
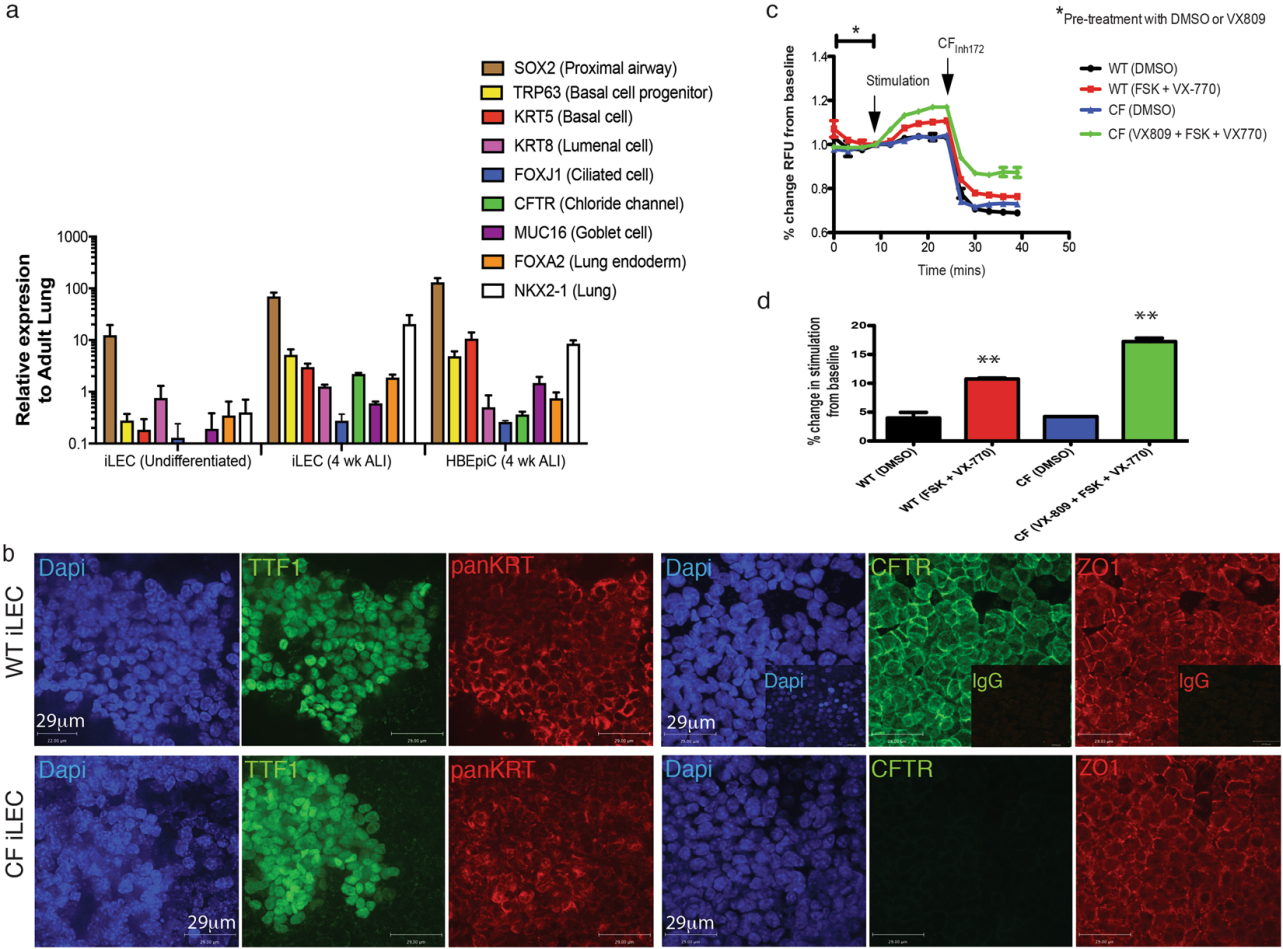
Pharmacological test of human iLEC-derived airway epithelium *in vitro*. (**a**) Human iLEC differentiated in air liquid interface culture for 4 weeks show up-regulated expression of lung endoderm (*NKX2-1*, *SOX2*, *FOXA2*) and specific lung epithelial cell (Basal: *KRT5*, *TRP63*, lumenal: *KRT8*, Ciliated: *FOXJ1* and Goblet: *MUC16*) genes in addition to the chloride channel *CFTR* similar to levels expressed by primary human bronchial epithelial cell (HBEpiC) cultures. N = 4 differentiated human iLEC lines. (**b**) Representative immunofluorescence characterization of wild-type iLEC cultured in ALI after 4 weeks show lung (TTF1+) epithelia (panKRT+) expressing tight-junction protein ZO1 and plasma membrane-localized CFTR. CF iLEC cultured in ALI after 4 weeks express similar airway epithelial markers but not plasma membrane-localized CFTR. Functional assessments were replicated n = 4 times on all four human iLEC lines. Scale bar represents 29 μm. (**c**) Representative line graph show change in fluorescence upon CFTR stimulation with 10 µM cAMP agonist Forskolin (FSK) and potentiator VX-770 followed by inhibition with CFTR specific inhibitor CFTRinh-172 in wild-type- and pharmacologically (VX-809) corrected CF iLEC-derived airway epithelia. (**d**) Bar graph reflects percent fold change in CFTR function from baseline levels. Biological replicates of ≥5 for each condition and t-test was performed (**p = 0.0084).

Further culture of the human iLECs for 4 weeks in air-liquid interface conditions, allowed up-regulated expression of several key lung epithelial cell markers including *NKX2-1*, *TRP63*, *KRT5*, *KRT8*, and *CFTR*, to levels similar to primary bronchial epithelial cells HBEpiC (Fig. 7a). Immunofluorescence characterization showed patches of NKX2-1-expressing epithelial cells (panKRT+). Patches of cells co-expressed plasma membrane-localized CFTR and the tight junction-coupled protein ZO1 (Fig. 7b). Importantly, *CFTR* mRNA expression was only observed in ALI culture conditions suggesting that ALI can induce further differentiation with up-regulated expression of airway epithelial markers and *CFTR* above baseline levels. Differentiated CF mutant (derived from CF fibroblasts harboring the deltaF508 mutation) iLEC in ALI cultures, also formed patches of NKX2-1 expressing epithelia. However, CFTR protein was not detected. To determine whether human iLEC in ALI exhibit functional CFTR activity, a modified apical chloride conductance (ACC) assay was performed as previously described^38^. Representative line-graph measurement of CFTR response over time showed functional CFTR conductance in wild-type iLEC upon stimulation with the agonist forskolin and CFTR modulator compound VX-770 (Fig. 7c). This response was specifically inhibited with CFTRinh-172 (p < 0.01). Correction of CFTR function in CF iLEC with a combination of the Vertex drug compounds VX-809 (corrector, Lumacaftor) and VX-770 (potentiator, Ivacaftor), together known as Orkambi®, an FDA-approved drug for the treatment of CF, showed significant CFTR-specific corrected function (p < 0.01, Fig. 7c,d). Thus, as a proof-of-concept, human iLEC can be used to screen for CFTR drug compounds and model CF disease *in vitro*.

## Discussion

Activation of the reprogramming factors (Oct4, Sox2, cMyc, and Klf4 or OSKM) in addition to the lung specifying transcription factor Nkx2-1, and lung-directed differentiation can generate induced lung epithelial-like cells (iLEC) from mouse embryonic and human dermal fibroblasts. The addition of the transcription factor Nkx2-1 led to early transcriptional changes towards lung epithelial phenotype suggesting the importance of Nkx2-1 in driving lineage specification of the cells towards lung cell fate. Interestingly, addition of the lung-specifying transcription factor Nkx2-1 concomitantly with OSKM did not yield lung epithelial phenotypes similar to Group A (Fig. 1d) suggesting that the cells must first lose the fibroblast phenotype and activate the epithelial program before specifying a lung transcriptional program. Over-expression of the lung specifying transcription factor Nkx2-1 alone did not induce any morphological epithelial-like changes or activate any lung-specific pathways supporting the idea that the pluripotency factors are necessary to induce a transient epigenetic state allowing exogenous inductive cues to trigger lineage-specific transdifferentiation/conversion^17,18^. Recent evidence have identified pivotal roles of lineage-specific transcription factors and microRNAs in inducing a permissive chromatin state during direct lineage reprogramming to enable chromatin accessibility, DNA methylation and lineage-specific gene expression^25,26^. The role of chromatin changes in this particular conversion protocol has yet to be determined.

In order to generate stable lung epithelial-like cells after transcription factor conversion of fibroblasts, growth factor-mediated signaling pathway activation was required, as has been previously shown with direct conversion into cardiomyocyte and neuronal cell lineages^17,18,39^. To do this, the cells were differentiated through a step-wise protocol^4^ mimicking lung development from the early definitive endoderm toward lung progenitors, using carefully timed treatments of growth factors and small molecule inhibitors. By the end of the conversion process, a small subset of epithelial-like cells emerged expressing the endogenous lung marker Nkx2-1 and Epcam. While Nkx2-1 is not solely expressed in the lung but also the thyroid and forebrain cell lineages, assessment of additional markers associated with the latter two cell types were not detected. A hallmark of iLEC is the ability to expand these cells in culture over multiple passages without losing the phenotype of the cells. Indeed, iLEC have maintained epithelial morphologies (>95% CD326+ cells) over several passages (>20 passages to date) and can survive freeze-thaw cycles. Overall, three steps 1. OSKM-mediated genomic and epigenomic changes leading to epithelial conversion, 2. Nkx2-1-mediated specification of the cells into lung precursors followed by, 3. Growth factor-mediated pathway activation of the lung program appears to be critical in the conversion of fibroblasts into lung epithelial-like cells.

Importantly, it should be noted that while isolating the cells based on CD326 expression does not yield a pure population of Nkx2-1 expressing cells, it does enrich for cells capable of forming mature airway epithelial cells when further differentiated. Human and mouse iLEC represent a heterogenous population of cells that can form airway structures with specialized lung cell types on decellularized lung scaffolds and after chimeric xenograft transplantation. While basal cell progenitors (P63+) were identified in both mouse and human iLEC, it remains unclear whether the multi-epithelial lineage differentiation potential resides in these basal cell progenitors or a result of selective enrichment of the epithelial cells from the heterogenous iLEC population using our assays. Future studies will need to identify methods to further isolate these progenitor cells within the CD326+ fraction and determine the precise phenotype of the cells with multi-epithelial lineage differentiation potential.

Paracrine signals from the extracellular matrix or supporting cells may play a role in the maturation of iLEC. Mouse iLEC were capable of repopulating decellularized rat lung scaffolds, but only when provided with rat fibroblast supporting cells. Similarly, when iLEC alone were injected subcutaneously into NOD-SCID mice in a chimeric teratoma assay, no airway structures or tumors were observed. However, when co-transplanted with embryonic stem cells, both mouse and human iLECs formed airway structures containing cells expressing Ciliated, Club, Basal, Goblet cell markers and proSPC+ cells. In the chimeric teratoma assay, human iLEC did not appear to contribute to other endoderm lineages such as the liver, thyroid, pancreas or gut and also did not contribute to the neuro-ectoderm lineage. Both mouse and human iLEC exhibited differentiation to both epithelial and supporting mesenchyme cells. This suggests that airway generation from iLEC requires mesenchymal cell support. Understanding the role of the mesenchymal paracrine signals that instruct lung lineage differentiation could improve the conversion efficiency and generate bona fide lung epithelial cell types. It remains unclear whether the cells forming the stromal cells derive from the subset of iLEC with continued expression of stromal markers as identified in the single cell analysis (Supplementary Fig. 4a). Future studies will need to address the heterogeneity of the iLEC and their differentiation potential into epithelial versus mesenchymal cell types.

It remains unclear if mouse and human iLEC are equivalent. Due to the heterogeneity in both human and mouse iLEC, it is difficult to compare the developmental potential of these cells. High expression of *Klf4* transgenes and endogenous *cmyc* in was observed in mouse but not human iLEC. While cmyc appear to be important in the development of bronchopulmonary progenitor cells^40^, and Klf4 regulates lung tumor development^41^, it remains unknown how continued expression of these factors in iLEC would impact cell conversion efficiency and downstream differentiation potential. It is likely that the varying conversion efficiency in both mouse and human cells may reflect the stochastic retroviral transduction of the factors, a caveat observed in viral-based reprogramming strategies.

This study cannot conclusively exclude the possibility that the converted iLEC transit through a pluripotency state such that the resulting iLEC are a product of directed differentiation from the transient iPS-like state, as has been previously described^19,20^. No expression of the endogenous pluripotency gene *Oct4/Pou5f1* was detected during the conversion process nor in the CD326+ iLEC. Similarly, *Sox2*, another transcription factor regulating pluripotency of embryonic stem cells, was not detected during the early stages of conversion but was expressed abundantly in iLEC. This suggests that the cells did not transit through a pluripotency state before differentiation into iLECs. In addition, the short timeline of conversion would suggest that complete reprogramming of fibroblasts into bona fide iPS cells prior to lung differentiation was unlikely.

An immediate and promising application of human iLEC is for CFTR drug testing and discovery *in vitro*. Here we show that CF (homozygous for deltaF508 mutation) iLEC-derived epithelia do not express functional CFTR protein but can be pharmacologically corrected with corrector (VX-809) and channel potentiator (VX-770) compounds. Given the demonstrated feasibility of generating and differentiating iLEC from different fibroblast lines harboring CF mutation within a relatively short time frame (~60 days) compared to the indirect route of generating induced pluripotent stem cells followed by differentiation of these cells towards lung (~4–6 months), iLEC may be an attractive cell source for rapid personalized drug screens. Although not the focus of this study, iLEC can potentially be used to evaluate other processes involved in CF lung disease such as bicarbonate secretion and the effects of other channels such as the sodium channel ENaC or other chloride channels. Future studies will need to further assess the full physiological functions of the cells. In light of recent evidence that the lung epithelium contains specialized ionocytes that express CFTR in relatively high abundance implicating their prominent role in CF^1^, future studies will also need to carefully assess the potential of iLEC to form these cell types.

Overall, this is the first study to demonstrate the potential to convert fibroblasts into stable lung-like epithelial cells in a rapid and reproducible method. With further refinement of the conversion process, these cells may provide an alternative cell source for generating functional lung cells for disease modeling and tissue regeneration.

## Materials and Methods

### Animals and human cell lines

Mice received care in compliance with the Principles of Laboratory Animal Care formulated by the National Society for Medical Research and the Guide for the Care and Use of Experimental Animals formulated by the Canadian Council on Animal Care. For mouse cell conversion studies, embryonic fibroblasts were isolated from embryos at day 15.5 of pregnancy from Nkx2-1-mCherry reporter mice^30^. For mice used in the teratoma or airway regeneration experiments, NOD.Cg-Prkdc^scid^Il2rg^tm1Wjl^ mice were either bred in-house or ordered from Jackson Laboratory at 4 weeks old. Mouse husbandry and manipulations were done in agreement with Canadian Council for Animal Care guidelines at the Toronto Centre for Phenogenomics or the University Health Network. Animal experiments were conducted according to the animal user protocol approved by Animal Care Committee of The Centre for Phenogenomics. The CA1 human embryonic stem cell line was approved for use by the Stem Cell Oversight Committee (SCOC) of CIHR and Research Ethics Board (REB) of The Hospital for Sick Children.

### Lentiviral expression plasmids

Human fibroblast cell lines GM00997, GM04320, GM01348A were obtained from the Coriell Cell repository (Coriell Institute for Medical Research) and SK0019-002 was obtained courtesy of Dr. James Ellis at the Hospital for Sick Children (Toronto, Canada). Before conversion, the human fibroblasts were infected with pLenti6/UbC/mSlc7a1 lentiviral vector (Addgene, 17224) expressing the mouse Slc7a1gene (encoding the receptor for ecotropic gamma retrovirus) and selected with blasticidin as previously described^3^.

### Cell conversion

In brief, retroviral vectors encoding mouse or human Oct-4, Sox2, Klf4 and c-Myc were produced using Plat-E cells by plasmid transfection of either pMXs-Oct4, pMXs-Sox2, pMXs-Klf4, and pMXs-c-Myc (Addgene plasmids 13366, 13367, 13370 and 13375 respectively) or pMXs-OCT4, pMXs-SOX2, pMXs-KLF4 and pMXs-CYMC (Addgene plasmids 17217, 17218, 17219 and 17220, respectively) as previously described^3^ Fibroblasts were seeded at 1 × 10^5^ cells/well onto 6 well plates and transduced once with the retroviral vectors and 8 μg/ml polybrene. The following day, the cells were allowed to recover in basic fibroblast media comprised of DMEM + 10% FBS + 1% penicillin/streptomycin (GIBCO). Two days post-retroviral transduction, mouse cells were infected with retroviral vector encoding MSCV-NKX2-1/PURO (Addgene plasmid 31271) and transduced cells enriched by puromycin-selection for 3 days. Human cells were transfected with pcDNA3.1(wt)-TTF1 (Addgene plasmid 49989) 2 days following viral transduction of OSKM.

Three days post-initial retroviral transduction, a step-wise differentiation process of the mouse fibroblasts and human fibroblasts was performed using a succession of defined media containing growth factors. Mouse fibroblasts were differentiated based on a previously published protocol to generate lung epithelia from mouse embryonic stem cells^31^. Human fibroblasts were differentiated based on our previously published protocol to generate lung epithelium from human pluripotent stem cells^4^. At least 3 independent experiments were performed for each mouse and human cell conversions. The converted cells were enriched by FACS for epithelial cell surface molecule CD326 and were subsequently called iLEC. While individual epithelial colonies were observed in the conversion process, no clonal isolation was performed due to the limited cell number per colony and increased cell death caused by mechanical disruption.

### Repopulation of decellularized rat lung scaffolds

Optimized procedures to decellularize the distal lungs of rats were adapted from previously published protocols^42^. Mouse iLEC (50,000 cells per group) were seeded onto 300- to 400-μm-thick sections of decellularized rat lung scaffolds along with 50,000 rat embryonic fibroblasts. Mouse iLEC were transfected with piggyBac GFP (pBCAG-GFP and pBase plasmids) using lipofectamine 3000 (Invitrogen) and FACS-sorted for GFP-high expression prior to the recellularization experiments. Cultures were maintained for 21 days on floating membranes (Nucleopore Track-Etched membranes, 8.0 μm pores) in a serum-free basal differentiation medium that was changed every other day. Cultures were maintained at 37 **°**C with 5% CO_2_. N = 4 per group.

### Chimeric teratomas

For teratoma assays, the mouse and human iLEC were transfected with piggyBac GFP (either pBCAG-GFP or pBCAG-H2B-GFP and pBase plasmids, courtesy of Dr. Andras Nagy, Samuel Lunenfeld Research Institute, Toronto) using lipofectamine 3000 (Invitrogen) and FACS-sorted for GFP-high expression prior to injection. All cells were washed with PBS once and suspended into 200 ul of 66% DMEM (Thermo Fisher CAT# 11960044) + 33% Matrigel Matrix High Concentration (Corning Cat#354248). The 200 ul mixture was injected subcutaneously into each dorsal flank of NOD-SCID gamma (NSG) mouse, (NOD-SCID IL2Rg^null^, JAX 005557). Palpable masses were formed 5–10 weeks after injection. To challenge the differentiation potential of iLEC, human (line CA1) or mouse (line R1) embryonic stem cells (ES) were co-transplanted with iLEC donors (1:4, 2 × 10^6^ ES: 8 × 10^6^ donor cells). As controls, 2.5 × 10^6^ human parental fibroblast or 8 × 10^6^ mouse embryonic fibroblast were suspended in 200 ul DMEM/matrigel mix, and injected subcutaneously into the contralateral dorsal flank of NSG mice. No palpable masses were formed during 10–16 weeks after injection. For mice with tumors, the animals were euthanized before the tumors reached endpoint size (1700 mm3), and tumors were dissected out and fixed in 10% formalin and paraffin-embedded prior to sectioning (8 μm thick) for H&E and IF staining. N = 2 for each cell line.

### Quantitative real-time PCR

Total RNA was prepared using the RNeasy Kit (Qiagen). RNA was reverse transcribed for first-strand cDNA using Superscript II (Invitrogen) according to manufacturer’s protocol. Quantitative real-time PCR (SYBR green detection method; Applied Biosystems, Foster City, CA) was performed for amplification of the genes. Real-time PCR (45 cycles of amplification) was performed on the LightCycler® 480 System (Roche). Gene expression was normalized to the housekeeping gene Gapdh and expressed relative to a positive control sample. Denaturing curves for each gene were used to confirm DNA product and eliminate possibility of pseudogene amplification or primer-dimers. All experiments were done in triplicate with at least 3 separate differentiation cultures. The comparative Ct method was employed (2^ΔΔCt^; ΔΔCt = ΔCtsample **− **ΔCtcalibrator). Relative gene expression values for calibrators were set to one (1), and values of target genes were represented as fold changes.

### Histology and immunofluorescence

Cells for characterization were seeded onto chamber slides and fixed with 4% PFA followed by staining for primary antibodies listed in Supplementary Table 1. Lung scaffolds from repopulation studies were embedded in HistoGel (Thermo Scientific) prior to fixation. Samples were processed according to standard conditions and sectioned at 5 μm. For paraffin-embedded sections including teratomas, deparaffinizations followed by rehydration of the sections were performed followed by antigen-retrieval using 10 mM citrate buffer, pH6.0. Sections were then allowed to cool, washed with PBS followed by blocking with buffer containing 2% BSA and 5% serum of the species in which the secondary antibody was raised. Primary antibody staining was performed overnight at 4 °C. For CFTR staining, transwells were fixed with ice-cold methanol (100%) in −20 °C for 10 minutes. For cytoplasmic or nuclear stains, cells were permeabilized and blocked with a solution containing 0.25% Triton-X100 (Invitrogen), 2% BSA and 5–10% normal goat or donkey serum prior to addition of primary antibodies. Secondary antibodies were performed at room temperature for 1 hour using goat anti-rabbit, mouse or rat (IgG,) or donkey anti-goat IgG Alexa Fluor 488 and 555, donkey anti-mouse, anti-rabbit, and anti-rat IgG conjugated to Alexa 546, 633 and 488 respectively and/or goat anti-chicken IgY Alexa Fluor 488 (all from Molecular Probes). Nuclei were counterstained with DAPI (Invitrogen). Images were visualized with the Confocal Digital Imaging System (Nikon) and analyzed with Volocity Software (PerkinElmer).

### Flow cytometry and FACS

Flow cytometry staining was performed as per manufacturer’s protocol with up to 10^6^ cells/sample. For intracellular staining, cells were fixed with 4% PFA followed by treatment with permeabilization (PERM) buffer containing saponin (BD Biosciences). Downstream primary and secondary antibody staining were performed in PERM buffer containing 2% BSA. For non-intracellular flow, the cells were resuspended and stained in FACS buffer containing 0.2% BSA. Primary antibodies used are listed in Supplementary Table 1. Secondary antibodies used include donkey anti-mouse, anti-goat and anti-rabbit IgG conjugated to Alexa Fluors 488, 546/555, or 633/647 (Molecular Probes). Non-immune reactive immunoglobulin isotypes were used as staining controls. Data acquisition was performed using the LSRII flow cytometer (BD Biosciences) and analyzed with FlowJo software (Tree Star Inc). Up to 5 × 10^4^ events were used for flow analysis. The Beckman MoFlo Astrios was used for FACS experiments.

### CFTR Membrane Potential Assay

Cells grown on transwell plates were analyzed for CFTR function using the apical chloride conductance (ACC) assay, as previously described^38^. The basal side had Hanks’ buffered solution containing chloride, and the apical solution contained chloride free buffer (150 mM NMDG-Gluconate, 3 mM K gluconate, 10 mM HEPES, pH 7.35, osmolarity 300 mOsm). The membrane potential dye (Molecular devices) was loaded in the apical compartment, at a concentration of 0.5 mg/ml. After 40 min of loading the dye at 37 °C, 5% CO_2_ and humidified air, the plate was transferred to the microplate reader (Molecular devices i3 multi-well microplate reader). Briefly, the reader was heated to 37 °C and the multi-point well scan was performed. CFTR was stimulated with cAMP agonist forskolin − 10 µM (Enzo life sciences) with or without CFTR potentiator VX-770 (Selleck). After maximal stimulation CFTR specific inhibitor CFTRinh-172 (CFFT) was used. Upon completion of experiment the data was exported in a tab-delimited format and analyzed.

### Immunoblotting

Cells were solubilized in 1% sodium dodecyl sulphate (SDS) and sample protein run on 6% SDS gels for SDS-polyacrylamide gel electrophoresis. Protein samples were transferred to nitrocellulose paper. Primary CFTR antibody (MAB1660 or antibody #450 courtesy of JR Riordan) and goat anti-mouse Ig HRP secondary antibody were used for immunoblotting. The immunoblot was exposed to enhanced chemiluminescence (ECL).

### Single cell gene expression analysis

Single propidium iodide negative mouse directly converted cells were FACS-sorted into 96 well plates for multiplex gene expression profiling using the 48 × 48 Dynamic array chip for the Fluidigm Biomark HD system (Fluidigm, San Francisco). Donor mouse cells that were Gfp+ and engrafted in the lungs were isolated and sorted directly into the 96 well plates for analysis. Cell lysis, reverse transcription and preamplification of the cDNA was done using the Thermo Scientific CellsDirect^TM^ One-step qRT-PCR kit. Analysis was done using R programming and the Fluidigm SINGuLAR Analysis Toolset v3.6.2. For control samples, Cd326 cell surface staining and mCherry fluorescence were used to enrich for adult and embryonic lung epithelial cells.

### Statistical analysis

Unless otherwise specified, for statistical analysis, unpaired t-tests were performed. When more than two groups were compared, one-way ANOVA was used followed by Dunnett’s post-test if significance was observed. Results were expressed as mean ± SEM.

## Supplementary information

Supplementary Files and Figures

## References

[1] Montoro DT (2018). A revised airway epithelial hierarchy includes CFTR-expressing ionocytes. Nature.

[2] Plasschaert LW (2018). A single-cell atlas of the airway epithelium reveals the CFTR-rich pulmonary ionocyte. Nature.

[3] Wong AP (2012). Directed differentiation of human pluripotent stem cells into mature airway epithelia expressing functional CFTR protein. Nat. Biotechnol..

[4] Wong AP (2015). Efficient generation of functional CFTR-expressing airway epithelial cells from human pluripotent stem cells. Nat Protoc.

[5] Firth AL (2015). Functional Gene Correction for Cystic Fibrosis in Lung Epithelial Cells Generated from Patient iPSCs. Cell Rep.

[6] McCauley Katherine B., Hawkins Finn, Serra Maria, Thomas Dylan C., Jacob Anjali, Kotton Darrell N. (2017). Efficient Derivation of Functional Human Airway Epithelium from Pluripotent Stem Cells via Temporal Regulation of Wnt Signaling. Cell Stem Cell.

[7] Chen Y-W (2017). A three-dimensional model of human lung development and disease from pluripotent stem cells. Nat. Cell Biol..

[8] de Carvalho Ana Luisa Rodrigues Toste, Strikoudis Alexandros, Liu Hsiao-Yun, Chen Ya-Wen, Dantas Tiago J., Vallee Richard B., Correia-Pinto Jorge, Snoeck Hans-Willem (2018). Glycogen synthase kinase 3 induces multilineage maturation of human pluripotent stem cell-derived lung progenitors in 3D culture. Development.

[9] Caiazzo M (2011). Direct generation of functional dopaminergic neurons from mouse and human fibroblasts. Nature.

[10] Vierbuchen T (2010). Direct conversion of fibroblasts to functional neurons by defined factors. Nature.

[11] Ieda M (2010). Direct reprogramming of fibroblasts into functional cardiomyocytes by defined factors. Cell.

[12] Miura S, Suzuki A (2017). Generation of Mouse and Human Organoid-Forming Intestinal Progenitor Cells by Direct Lineage Reprogramming. Cell Stem Cell.

[13] Kaminski MM (2016). Direct reprogramming of fibroblasts into renal tubular epithelial cells by defined transcription factors. Nat. Cell Biol..

[14] Huang P (2014). Direct reprogramming of human fibroblasts to functional and expandable hepatocytes. Cell Stem Cell.

[15] Szabo E (2010). Direct conversion of human fibroblasts to multilineage blood progenitors. Nature.

[16] Li J (2013). Conversion of human fibroblasts to functional endothelial cells by defined factors. Arterioscler. Thromb. Vasc. Biol..

[17] Efe JA (2011). Conversion of mouse fibroblasts into cardiomyocytes using a direct reprogramming strategy. Nat. Cell Biol..

[18] Kim J (2011). Direct reprogramming of mouse fibroblasts to neural progenitors. Proc. Natl. Acad. Sci. USA.

[19] Bar-Nur O (2015). Lineage conversion induced by pluripotency factors involves transient passage through an iPSC stage. Nat. Biotechnol..

[20] Maza I (2015). Transient acquisition of pluripotency during somatic cell transdifferentiation with iPSC reprogramming factors. Nat. Biotechnol..

[21] Koche RP (2011). Reprogramming factor expression initiates widespread targeted chromatin remodeling. Cell Stem Cell.

[22] Fussner E (2011). Constitutive heterochromatin reorganization during somatic cell reprogramming. EMBO J..

[23] Hewitt KJ, Garlick JA (2013). Cellular reprogramming to reset epigenetic signatures. Mol. Aspects Med..

[24] Mattout A, Biran A, Meshorer E (2011). Global epigenetic changes during somatic cell reprogramming to iPS cells. J Mol Cell Biol.

[25] Abernathy DG (2017). MicroRNAs Induce a Permissive Chromatin Environment that Enables Neuronal Subtype-Specific Reprogramming of Adult Human Fibroblasts. Cell Stem Cell.

[26] Wapinski OL (2017). Rapid Chromatin Switch in the Direct Reprogramming of Fibroblasts to Neurons. CellReports.

[27] Zhu S (2016). Human pancreatic beta-like cells converted from fibroblasts. Nat Commun.

[28] Cao N (2016). Conversion of human fibroblasts into functional cardiomyocytes by small molecules. Science.

[29] Zhang M (2016). Pharmacological Reprogramming of Fibroblasts into Neural Stem Cells by Signaling-Directed Transcriptional Activation. Cell Stem Cell.

[30] Bilodeau M, Shojaie S, Ackerley C, Post M, Rossant J (2014). Identification of a proximal progenitor population from murine fetal lungs with clonogenic and multilineage differentiation potential. Stem Cell Reports.

[31] Mou H (2012). Generation of multipotent lung and airway progenitors from mouse ESCs and patient-specific cystic fibrosis iPSCs. Cell Stem Cell.

[32] Rock JR (2009). Basal cells as stem cells of the mouse trachea and human airway epithelium. Proc. Natl. Acad. Sci. USA.

[33] Rock JR (2011). Notch-dependent differentiation of adult airway basal stem cells. Cell Stem Cell.

[34] Vaughan AE (2015). Lineage-negative progenitors mobilize to regenerate lung epithelium after major injury. Nature.

[35] Yamaguchi T, Hosono Y, Yanagisawa K, Takahashi T (2013). NKX2-1/TTF-1: an enigmatic oncogene that functions as a double-edged sword for cancer cell survival and progression. Cancer Cell.

[36] Hotta A (2009). EOS lentiviral vector selection system for human induced pluripotent stem cells. Nat Protoc.

[37] Wang X-Y (2012). Novel method for isolation of murine clara cell secretory protein-expressing cells with traces of stemness. PLoS ONE.

[38] Ahmadi S (2017). Phenotypic profiling of CFTR modulators in patient-derived respiratory epithelia. npj Genomic Med.

[39] Zhu S, Wang H, Ding S (2015). Reprogramming fibroblasts toward cardiomyocytes, neural stem cells and hepatocytes by cell activation and signaling-directed lineage conversion. Nat Protoc.

[40] Broers JL (1993). Expression of c-myc in progenitor cells of the bronchopulmonary epithelium and in a large number of non-small cell lung cancers. Am J Respir Cell Mol Biol.

[41] Yu T (2016). KLF4 regulates adult lung tumor-initiating cells and represses K-Ras-mediated lung cancer. Cell Death Differ..

[42] Shojaie S (2015). Acellular lung scaffolds direct differentiation of endoderm to functional airway epithelial cells: requirement of matrix-bound HS proteoglycans. Stem Cell Reports.

