# Supplementary material for: Conversion of human and mouse fibroblasts into lung-like epithelial cells
Source: Sci Rep. 2019 Jun 21;9:9027. doi: 10.1038/s41598-019-45195-y (PMC6588580; doi:10.1038/s41598-019-45195-y)

Conversion of human and mouse fibroblasts into lung-like epithelial cells

Amy P. Wong, Sharareh Shojaie, Qin Liang, Sunny Xia, Michelle Di Paola, Saumel Ahmadi, Claudia Bilodeau, Jodi Garner, Martin Post, Pascal Duchesneau, Thomas K. Waddell, Christine E. Bear, Andras Nagy, Janet Rossant

Supplementary Files and Figures

Supplementary Figures

Figure S1. (a) Immunofluoresence staining for Ttf1 (*Nkx2-1*) show colocalization of the protein with mCherry transgene in a colony (representative white hatched area) of converted mouse cells and (b) day 1 post Cd326 sort (representative yellow hatched area). (c) G-banding karyotype analysis of the iLEC. (d) Real-time qRT-PCR analysis of gut, liver, thyroid and forebrain lineages in mouse iLEC and other control tissues. Genes were normalized to housekeeping gene Gapdh and expressed relative to respective control tissues. N=3 independent mouse iLEC conversion characterization. (e) Endogenous and transgene analysis of conversion factors in mouse converted cells. Genes were normalized to housekeeping gene Gapdh and expressed relative to respective control (black bar).

Figure S2. (a) Alternative conversion strategy for mouse fibroblasts. (b) Table summarizing the conversion efficiency when the cells were transduced with Nkx2-1. EMT; epithelial-mesenchymal transition, NA; not available. (c) Representative fluorescence (top) and DIC (bottom) images of epithelial-like colonies at the end of the conversion process. With the exception of Group A transduced with OSKM alone, Group B and iLEC showed epithelial-like colonies with mCherry expression. Note: Group B, with the highest mcherry expressing colonies expressed high levels *Nkx2-1* transgene. Yellow hatched area outlines mcherry expression in the respective conversion group. (d) Endogenous and transgene analysis of conversion factors in Group B cells that expressed the strongest mcherry transgene. N=3 independent mouse iLEC conversion characterization. Scale bar represents 150μm.

Figure S3. (a) Representative image of mouse iLEC under low magnification. Mixed non-immune immunoglobulins (IgG) were used to exclude non-specific binding of secondary antibodies. (b) Characterization of mouse iLEC after 1 passage show mouse iLEC do not express distal epithelial cell markers proSPC or Ccsp. Scale bars represent 90-100μm.

Figure S4. Unbiased hierarchical clustering of single cells from all three groups show single iLEC segregate into 3 distinct groups. Most of the iLEC (38/48) do not cluster with either adult lung epithelial cells or embryonic lung epithelia (green box). These cells demonstrate up-regulated expression of lung developmental genes such as *Gata6* and *Pdpn* but also foregut endoderm markers *Pax9*, *Otx2* and early mesenchymal marker *Snail*. Few 6/48 iLEC clustered with both embryonic and adult lung epithelial cells (black box) and expressed many of the lung genes (see manuscript Fig 2f). Interestingly 4/48 iLEC (blue box) clustered with some adult lung epithelial cells and appear to express high levels of *Epcam* and down-regulated expression of lung developmental genes *Gata6* and *Pdpn*.

Figure S5. (a) Representative global (low magnification) view of the recellularized rat lung scaffolds with mouse iLEC (Gfp+) show airway recellularization with lung epithelia (Ttf1+panKrt+) cells. (b) Representative H&E images of recellularized scaffolds showing a mass of Group B cells in the decellularized graft (hatched). (c) Immunofluorescence characterization show Group B donor (Gfp+) cells that express lung marker Ttf1 but not epithelial (panKrt-negative, yellow hatched area). Scale bars represent 45-200μm.

Figure S6. (a) Representative hematoxylin & eosin staining of mouse teratoma formed by R1 ES cells alone. (b) Immunofluorescence staining of mouse teratomas for various lung epithelial cell lineages. Three independent batches of R1 ES cells were used to generate 6 teratomas in 3 transplanted mice recipients. Scale bars represent 22-45μm. Non-immune immunoglobulins (IgY and IgGs) were used to exclude non-specific binding of secondary antibodies. Yellow hatched represent areas of mouse ES-derived epithelia.

Figure S7. Normal mouse lung was used as staining controls. Immunofluorescence staining for various lung epithelial cell markers. Scale bar represents 22μm. Non-immune immunoglobulins (IgY and IgGs) were used to exclude non-specific binding of secondary antibodies.

Figure S8. (a) Representative DIC images of additional human iLEC derived from fibroblasts harboring the Cystic Fibrosis main mutation F508del genotype. Epithelial colonies emerged during the conversion process (yellow hatched borderline). FACS-sorting for CD326 enriched for epithelial cells. Scale bar represents 150-200μm. (b) Human bronchial epithelial cells were used as controls for immunofluorescence characterization of the iLEC. Scale bar represents 45μm.

Figure S9. Additional characterization of iLEC (GFP+) contribution to the chimeric teratomas. (a) Low magnification teratoma images show wider view of donor GFP+ iLEC contribution to both airway (white arrows) and non-airway structures (yellow arrows). (b) GFP+ iLEC do not contribute to pancreas, liver, thyroid, forebrain lineages. Cells positive for the respective markers (yellow arrows) were GFP-negative suggesting they were ES cell-derived. Scale bars represent 45-120μm.

Figure S10. (a) Characterization of human ES cell-derived teratomas. (b) Human tracheal and lung tissues were used as experimental and staining controls respectively. Immunofluorescence staining for various lung epithelial cell markers. White hatched represent areas of human ES-derived epithelia. White bar represents 29μm. Non-immune immunoglobulins (IgGs) were used to exclude non-specific binding of secondary antibodies.

Supplementary Table 1: List of Antibodies

Antibody	Clone	Manufacturer	ID	Assay	Dilution
panKRT	AE1/AE3	Abcam	Ab80826	IF,FC	1:350
panKRT	AE1/AE3	Dako	Z0622	IF	1:500
Krt14	LL002	Abcam	Ab7800	IF	1:1000
Krt8/18	EP1628Y	Abcam	Ab53280	IF	1:500
Krt5		Abcam	Ab53121	IF	1:500
Krt19	RCK108	Dako	M0888	IF	1:400
CFTR	L12B4	Millipore	MAB3484	IF	1:250
CFTR	MM13-4	Millipore	05-581	IF	1:100
CCSP		Abcam	Ab40873	IF	1:300
CCSP	T-18	Santa Cruz	Sc-9772	IF	1:200
NKX2-1	EP1584Y	Abcam	Ab76013	IF,FC	1:500
NKX2-1	8G7G3/1	Dako	M3575	IF,FC	1:300
ZO1		Invitrogen	33-9100	IF	1:100
SOX2		R&D Systems	AF2018	IF,FC	1:250
SOX2	N1C3	GeneTex	GTX101507	IF	1:500
SOX17	245013	R&D Systems	MAB1924		1:100
deltaN P63	Poly6190	BioLegend	619002	IF,FC	1:500
P63	EPR5701	Abcam	Ab124762	IF	1:100
POUF51		GeneTex	GTX100622	IF, FC	1:500
Thyroglobulin	EPR9730	Abcam	Ab156008	IF	1:200
Ki67	MIB-5	Dako	M7248	IF	1:200
GFP		Abcam	Ab13970	IF	1:200
Fsp1	S100A4	Millipore	07-2274	FC	1:250
FOXJ1	2A5	eBioscience	14-9965	IF	1:100
FOXA2	M-20	Santa Cruz	Sc-6554	IF	1:100
Acetylated a-tubulin	6-11B-1	Abcam	Ab24610	IF	1:300
ProSFTPC		Abcam	Ab40879	IF	1:250
SCGB3A2	K-12	Santa Cruz	Sc-48320	IF	1:250
Vimentin	EPR3776	Abcam	Ab92547	IF	1:250
Muc5ac	45M1	ThermoFisher	MA5-12178	IF	1:200
Muc5b	19.4E	Abcam	Ab77995	IF	1:200
Muc1	NCRC48	Abcam	Ab28081	IF	1:250
Nestin	10C2	Millipore	MAB5326	IF	1:300
Hpd1	DHIC2-4A10	Stemgent	09-0046	IF	1:250
PDX1	A-17	Santa Cruz	Sc-14664	IF	1:200
B3-tubulin	2G10	Sigma	T8578	IF	1:300
PAX6	AD2.38	Abcam	Ab78545	IF	1:250
PAX8	EPR13511	Abcam	Ab189249	IF	1:250
HNF6		Abcam	Ab83564	IF	1:200
CDX2	EPR2764Y	Abcam	Ab76541	IF	1:200
Albumin	HSA-11	Sigma	SAB4200711	IF	1:500
CD326-A488	9C4	BioLegend	324210	FC	1:500

Supplementary Fig 1

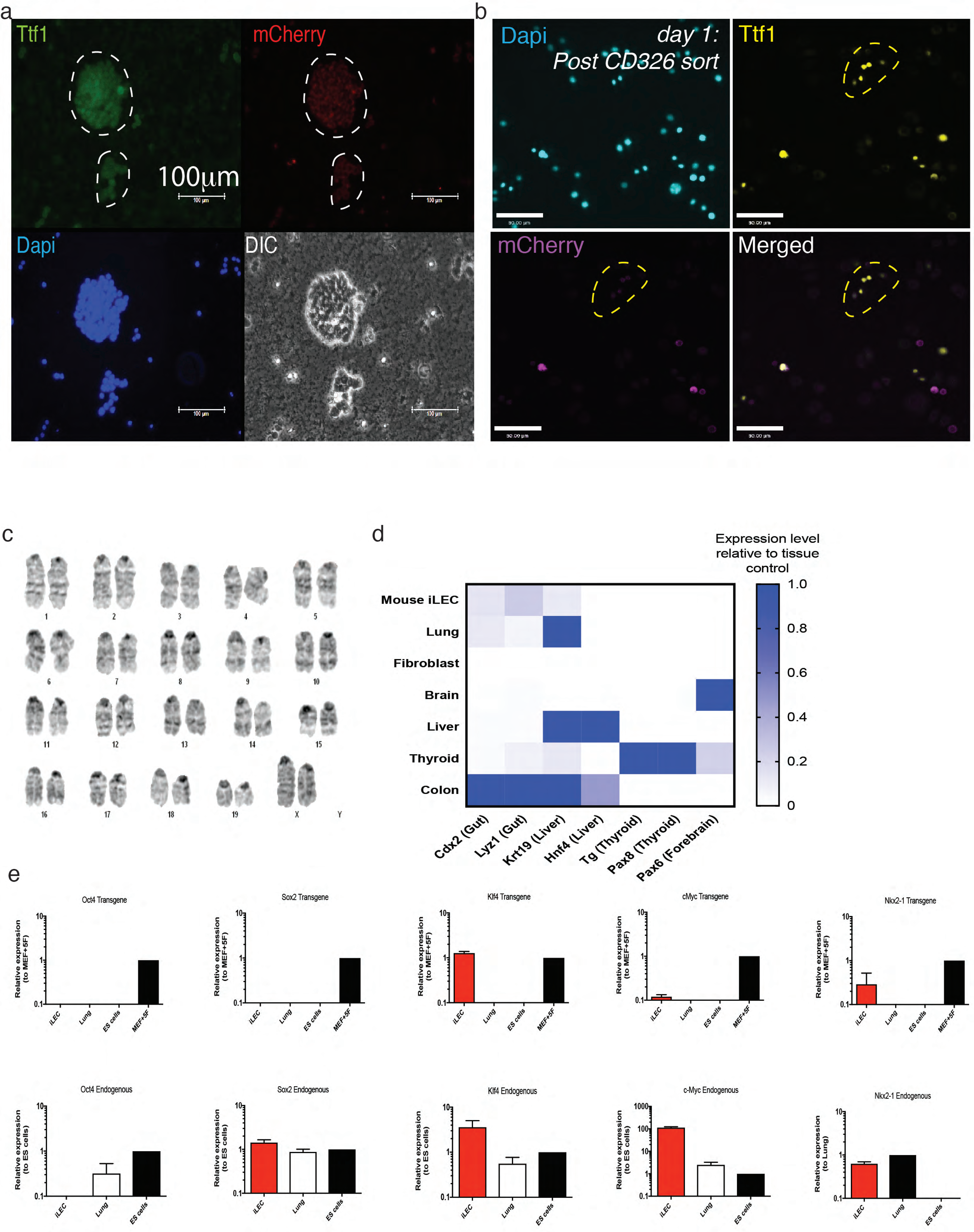

Supplementary Fig 2

a

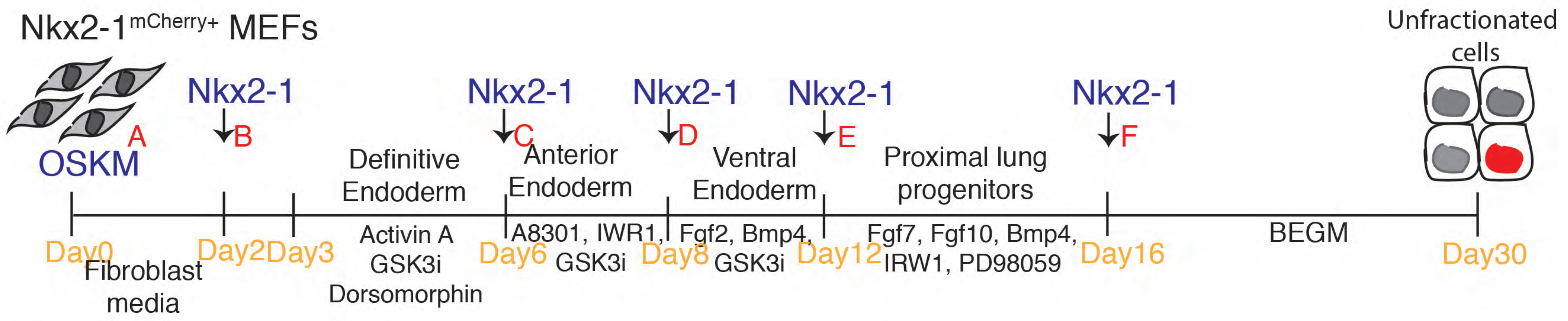

b

Conditions	Starting cell population	Conversion ?	% Epcam+ cells	Morphology after extended culture
A: OSKM → BEGM	Fibroblast	No	NA	NA
B: OSKM → Nkx2-1 → BEGM	Fibroblast	Yes		
C: OSKM→ 3 days in DE → Nkx2-1 → BEGM	Definitive endoderm	No	NA	NA
D: OSKM → 3 d in DE + 2 d anteriorization → Nkx2-1 → BEGM	Anterior endoderm	No	NA	NA
E: OSKM → 3 d in DE + 2 d anteriorization + 4 d ventralization → Nkx2-1 → BEGM	A/V endoderm	Yes	63.4%	EMT
F: OSKM → 3 d in DE + 2 d anteriorization + 4 d ventralization + 4 d Lung specification → Nkx2-1 → BEGM	Lung progenitors	Yes	<0.1%	pseudo-fibroblast-like

*NA = not applicable; EMT = epithelial-mesenchymal transition

c

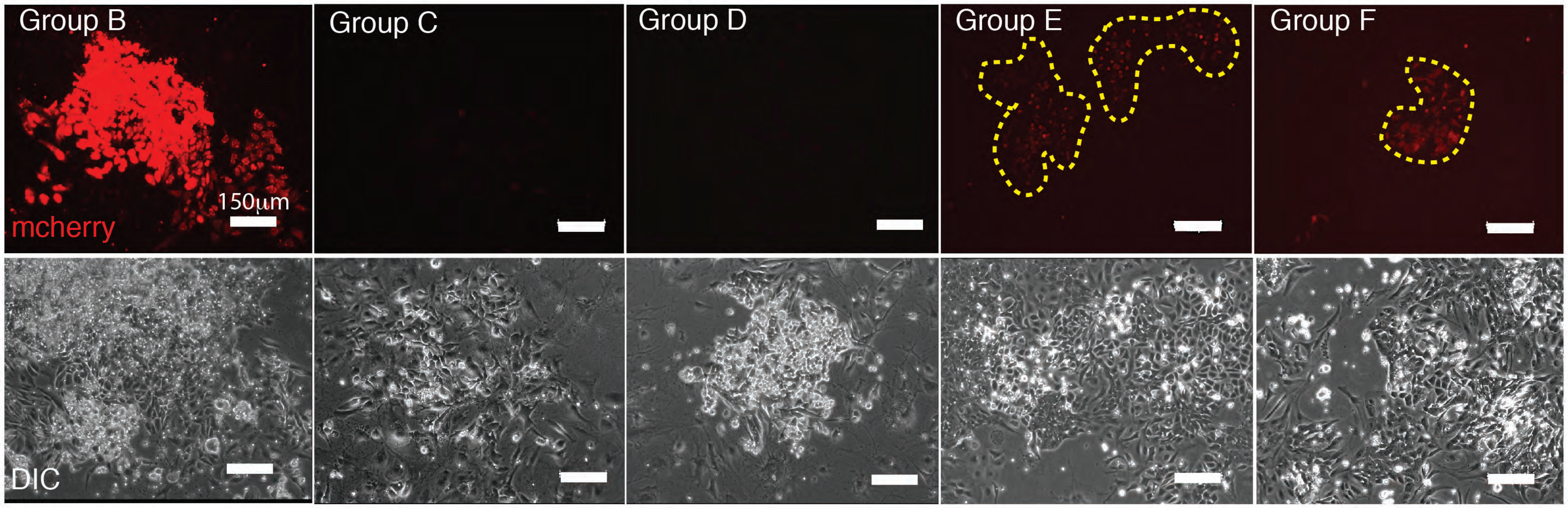

d

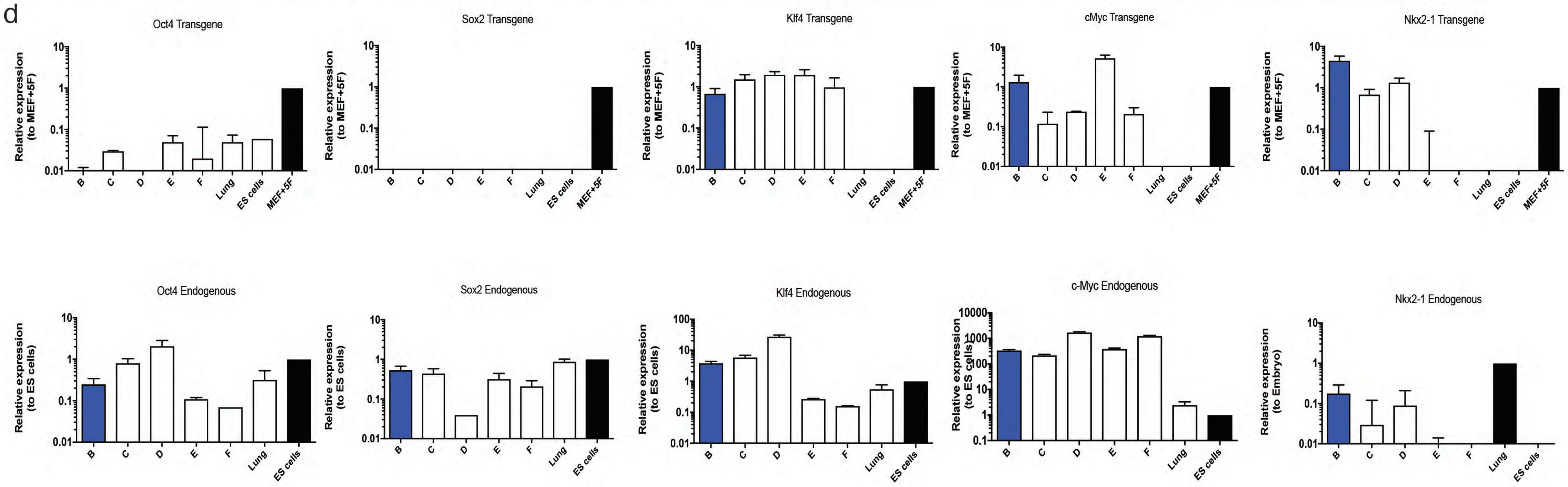

Supplementary Fig 3

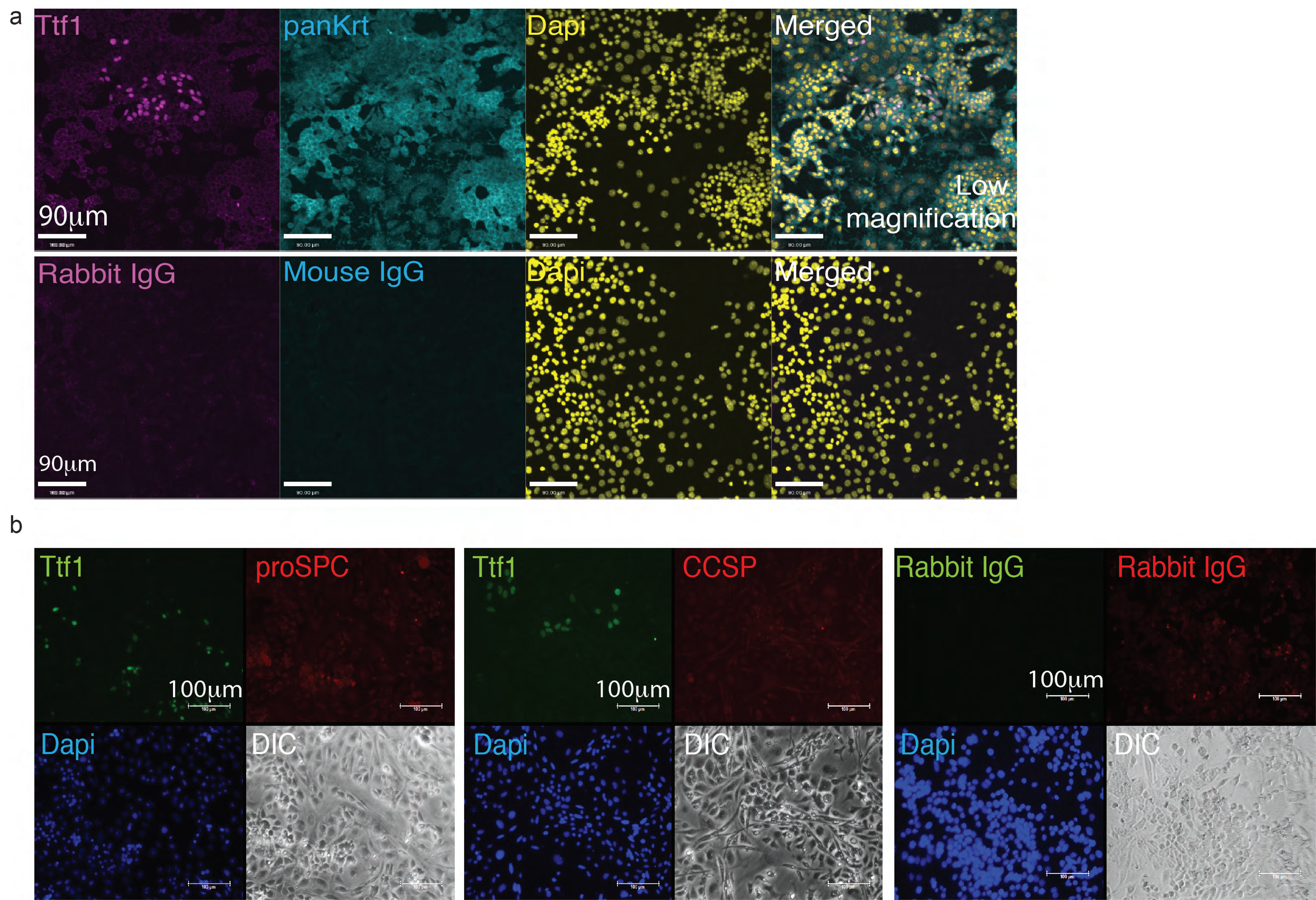

Supplementary Fig 4

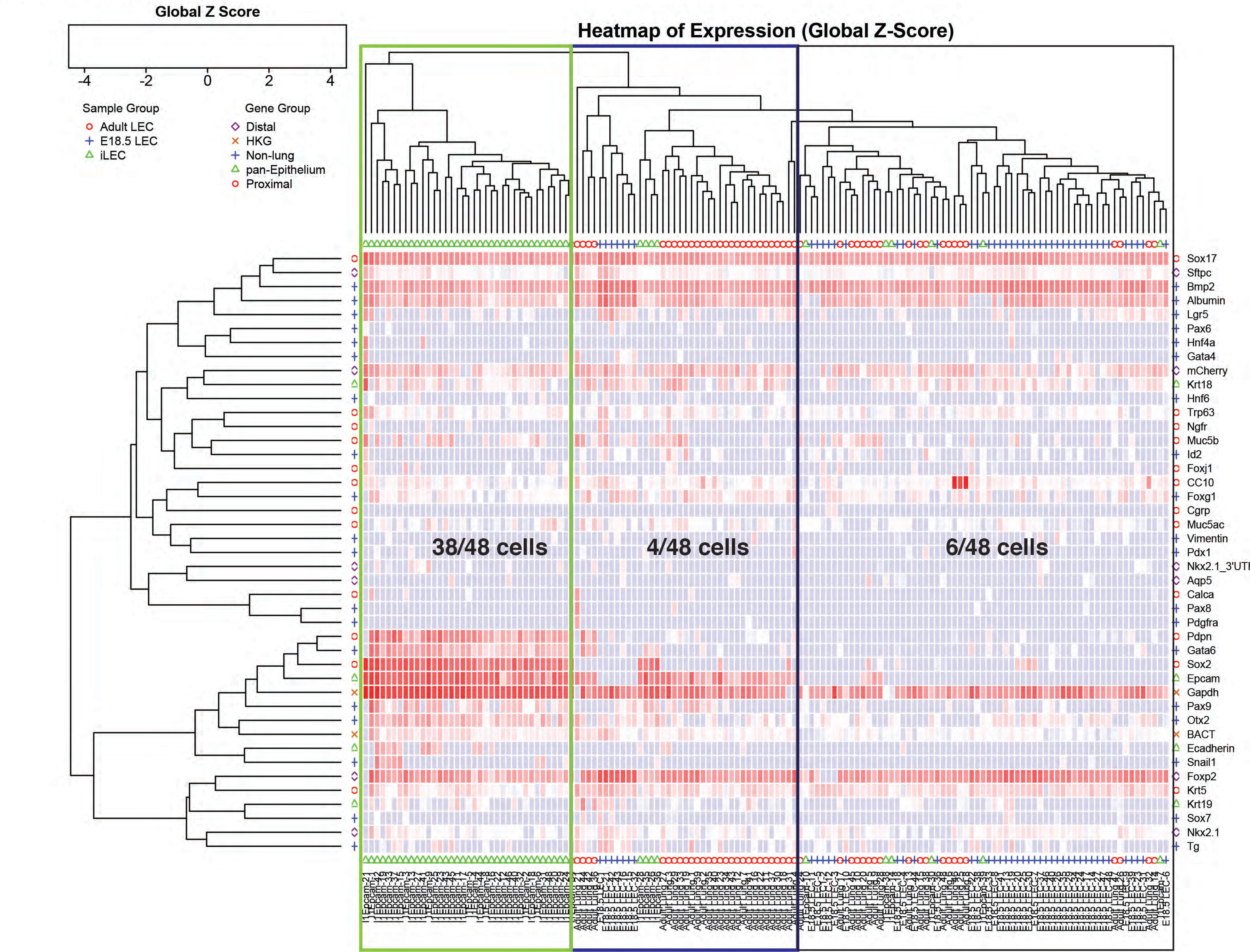

Supplementary Fig 5

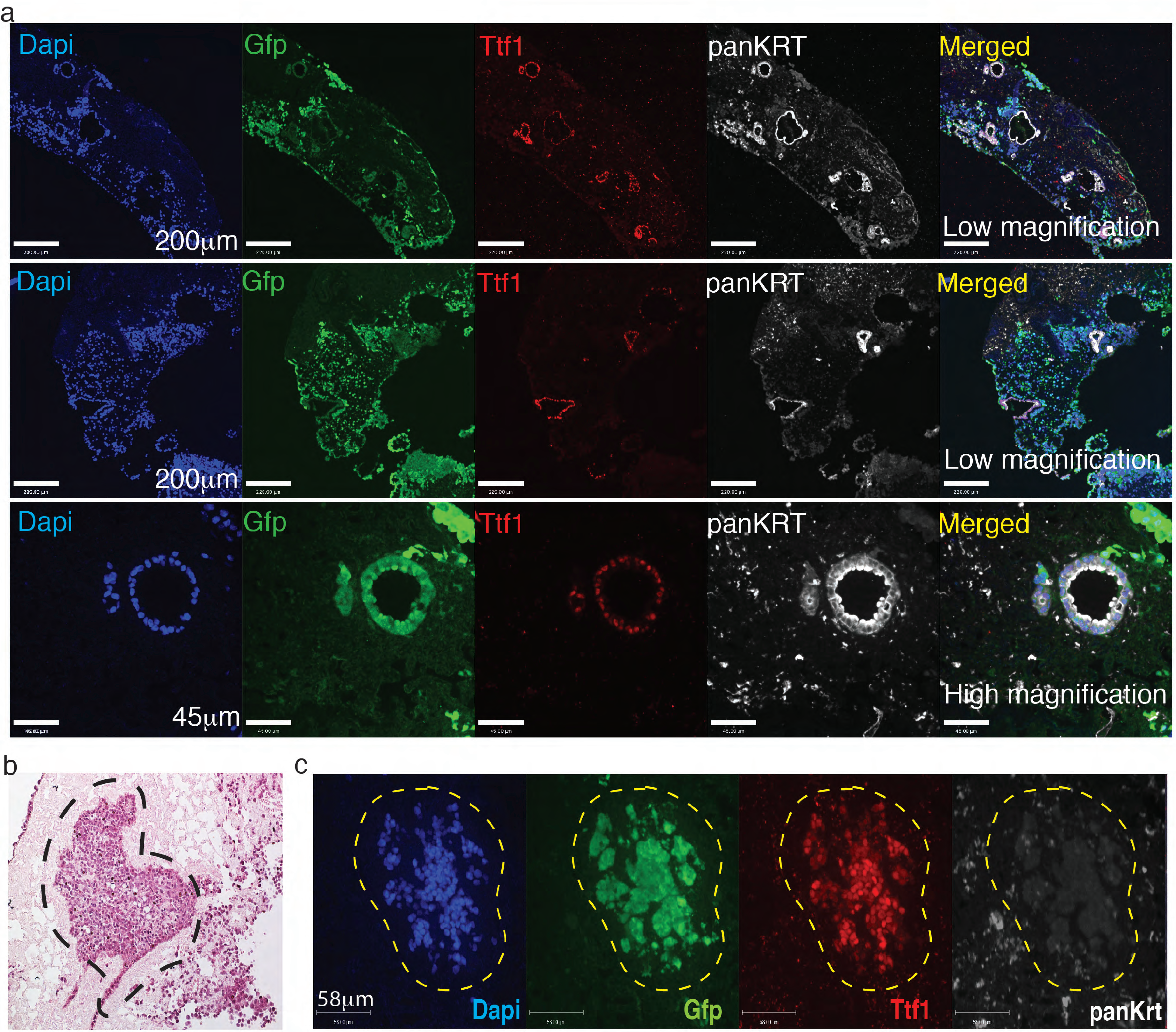

Supplementary Fig 6

a

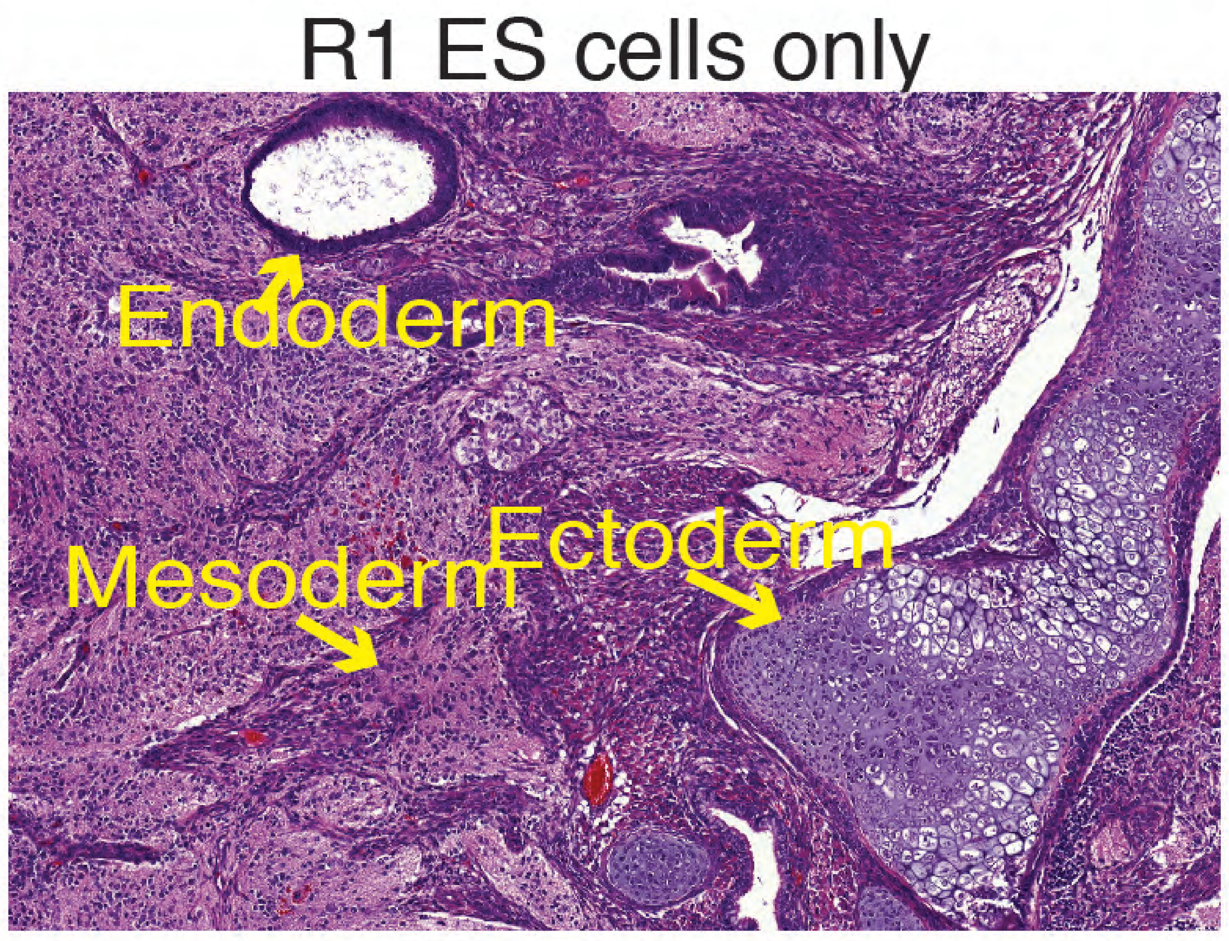

b

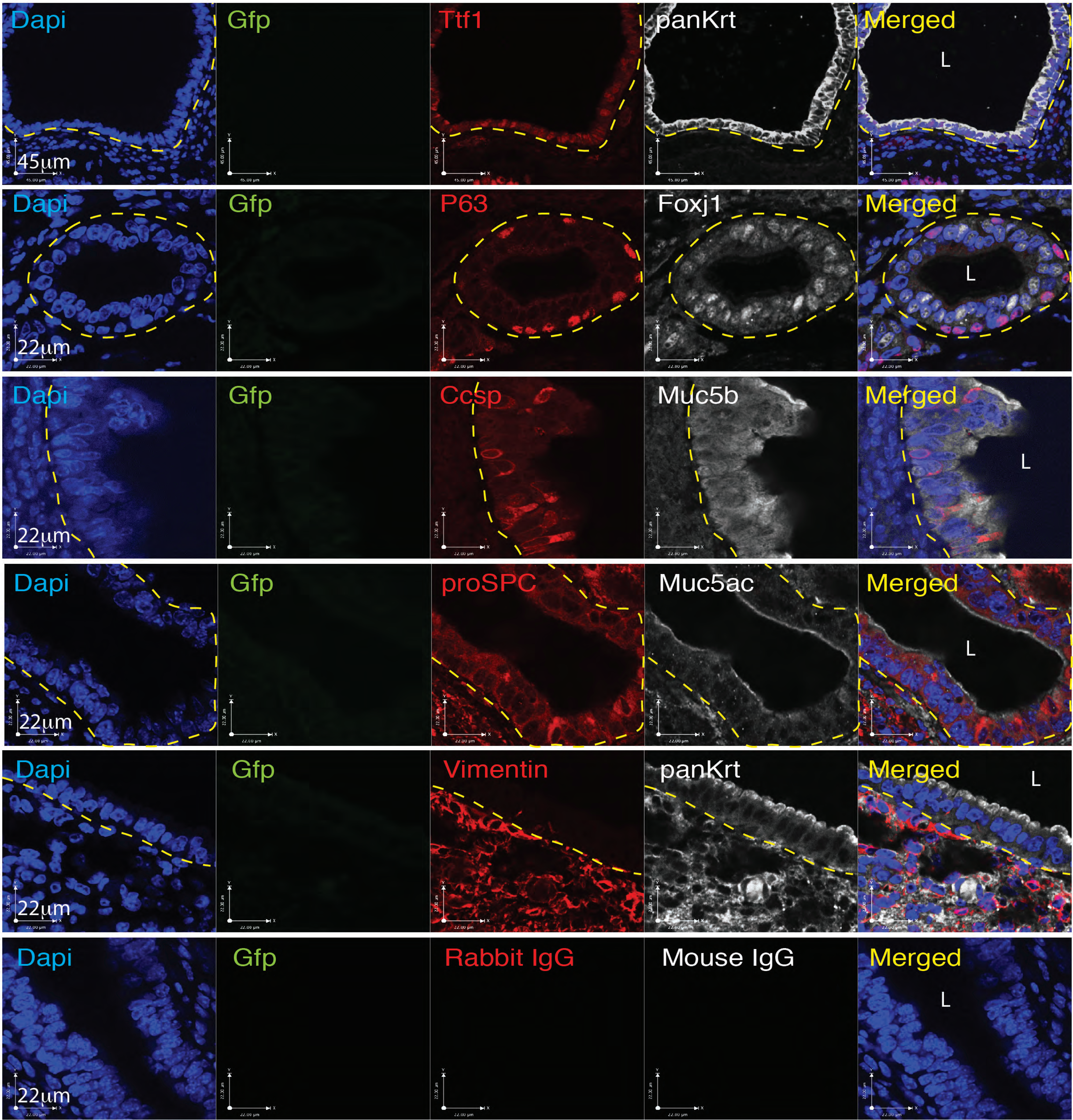

Supplementary Fig 7

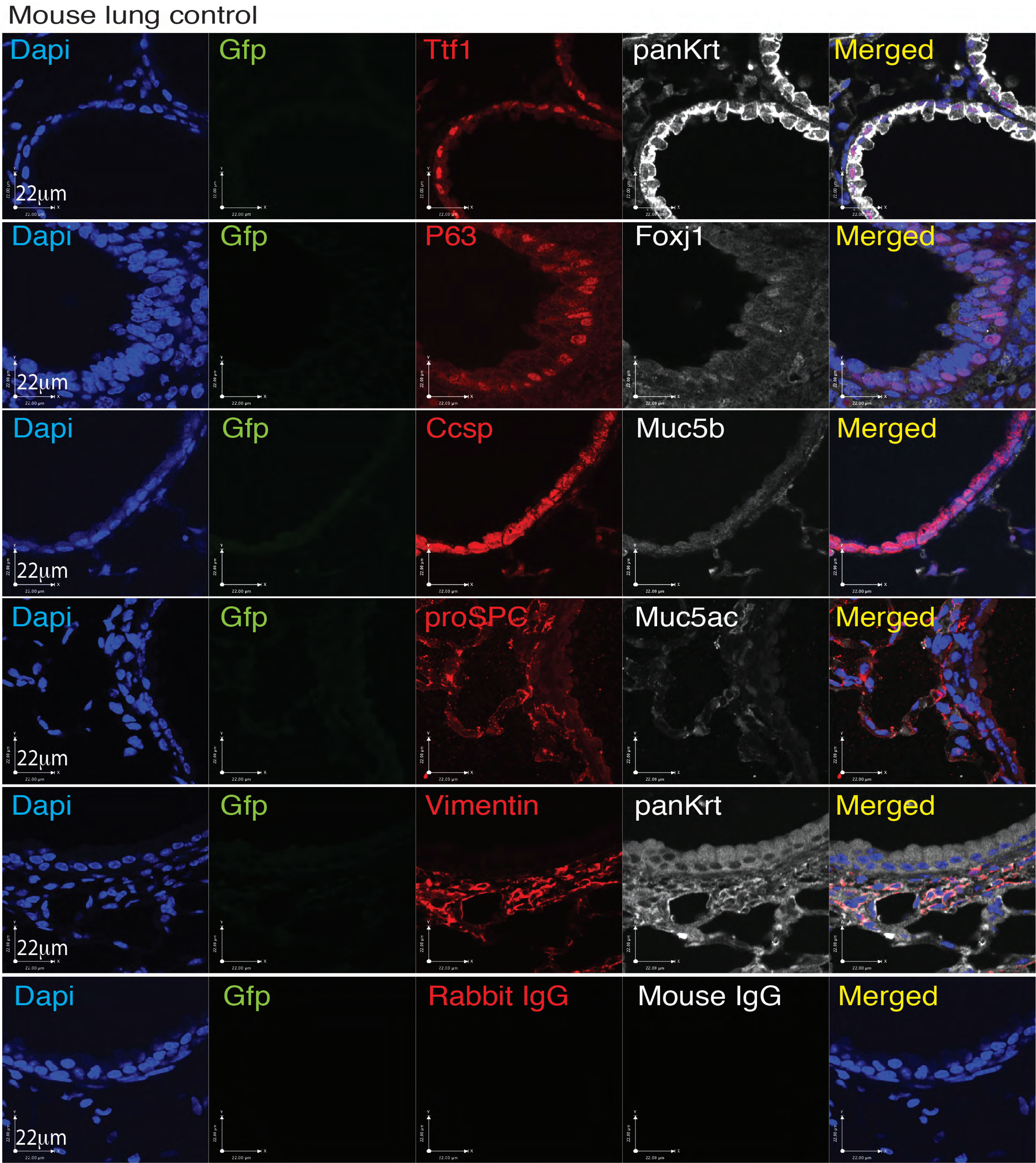

Supplementary Fig 8

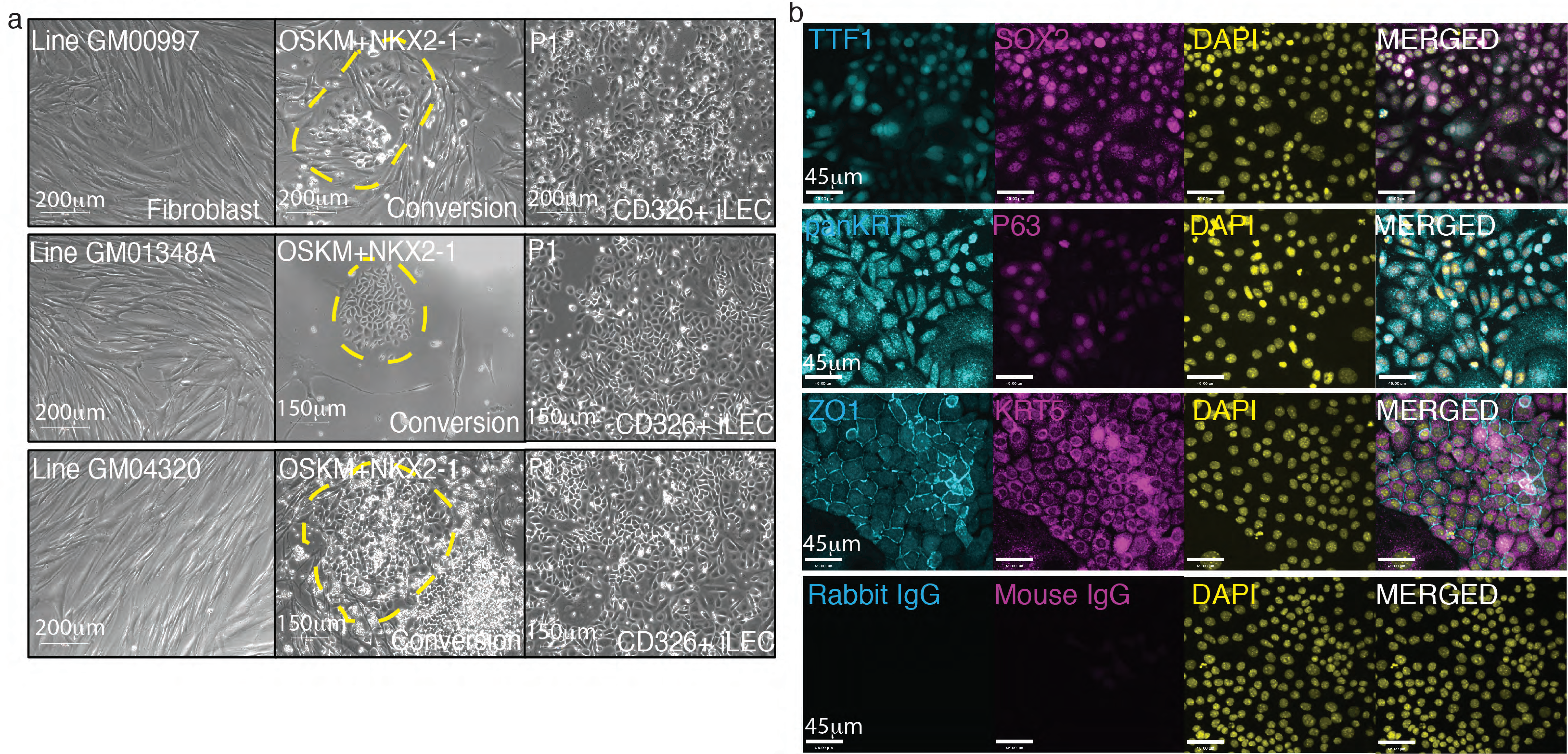

Supplementary Fig 9

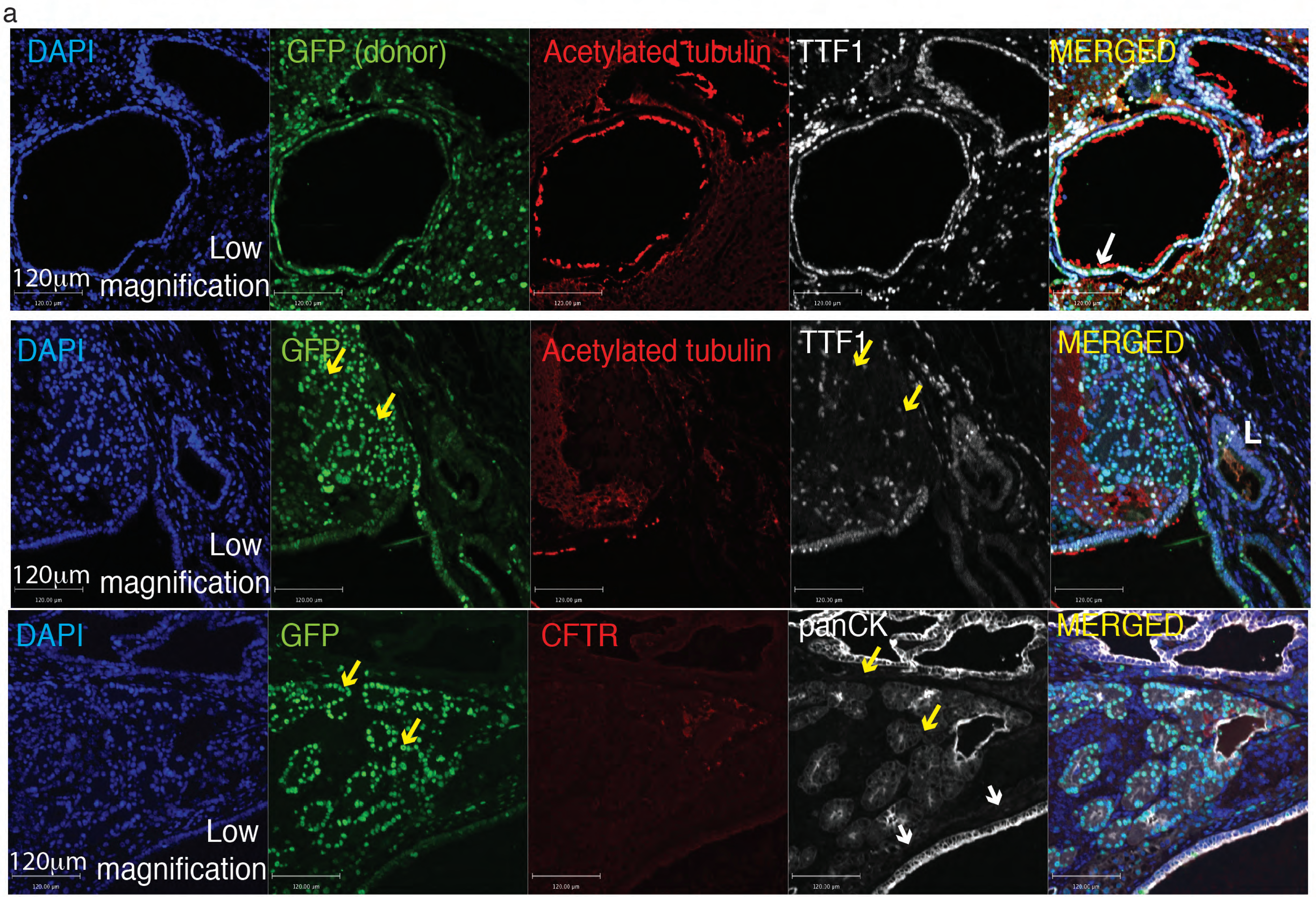

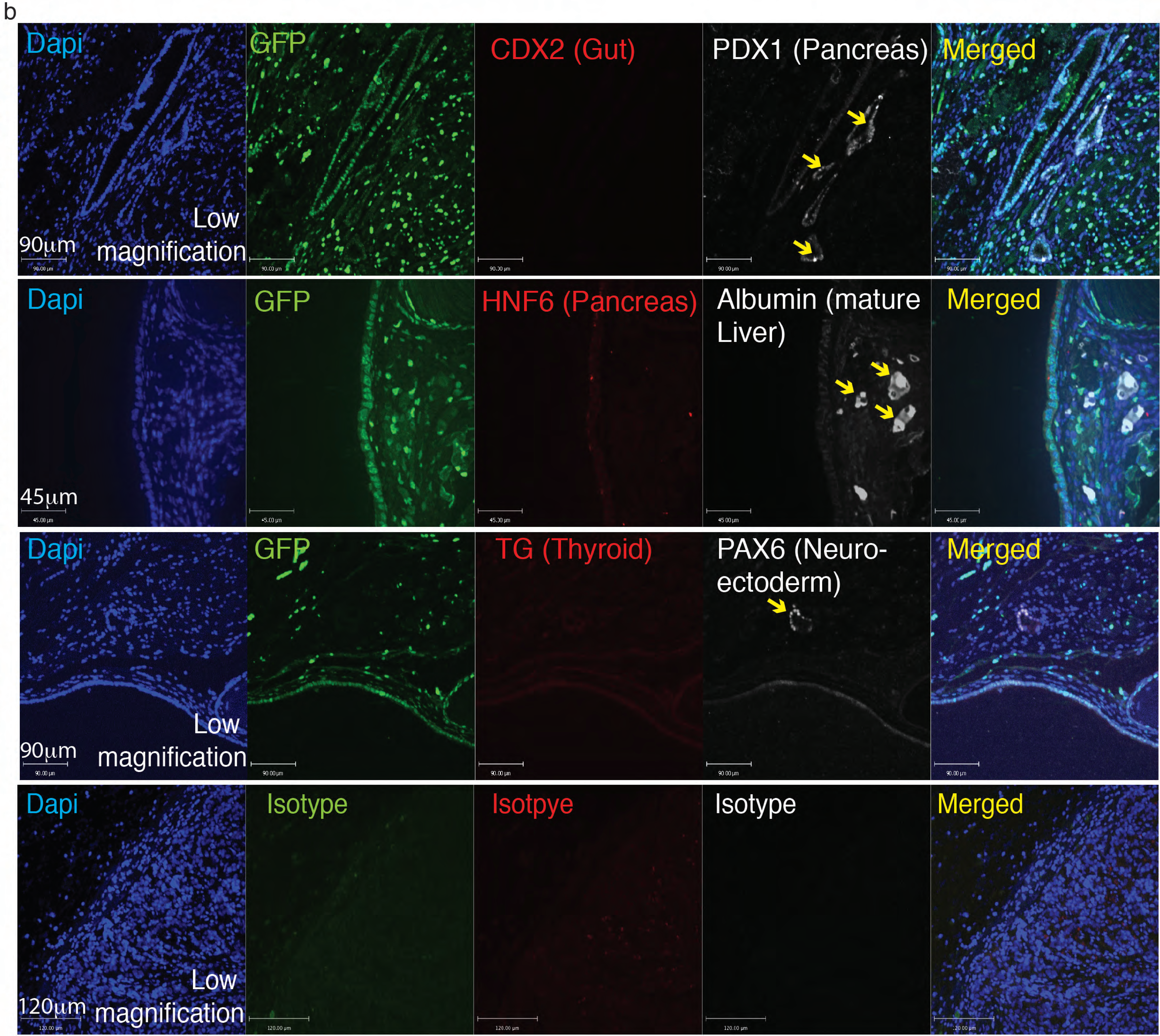

Supplementary Fig 10

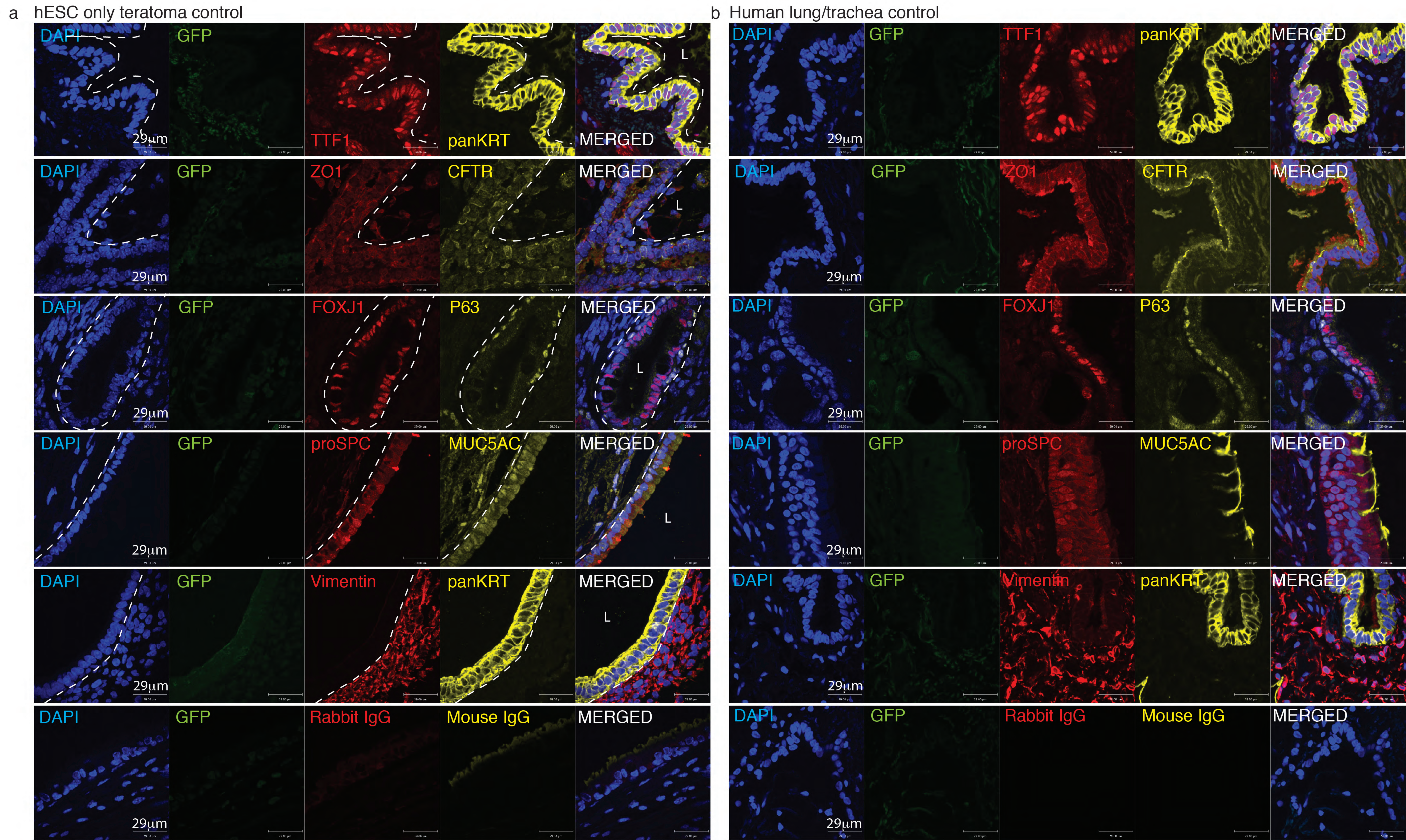

Supplement: Supplementary file 1 — Supplementary Files and Figures [file 41598_2019_45195_MOESM1_ESM.pdf]
